# The Human Leukocyte Antigen-DPB1 Degree of Compatibility Is Determined by Its Expression Level and Mismatch Permissiveness: A German Multicenter Analysis

**DOI:** 10.3389/fimmu.2020.614976

**Published:** 2021-01-25

**Authors:** Daphne Mytilineos, Chrysanthi Tsamadou, Christine Neuchel, Uwe Platzbecker, Donald Bunjes, Natalie Schub, Eva Wagner-Drouet, Gerald Wulf, Nicolaus Kröger, Niels Murawski, Hermann Einsele, Kerstin Schaefer-Eckart, Sebastian Freitag, Jochen Casper, Martin Kaufmann, Mareike Dürholt, Bernd Hertenstein, Stefan Klein, Mark Ringhoffer, Carlheinz R. Mueller, Sandra Frank, Hubert Schrezenmeier, Daniel Fuerst, Joannis Mytilineos

**Affiliations:** ^1^ Institute of Clinical Transfusion Medicine and Immunogenetics Ulm, German Red Cross Blood Transfusion Service, Baden Wuerttemberg-Hessen, and University Hospital Ulm, Ulm, Germany; ^2^ Institute of Transfusion Medicine, University of Ulm, Ulm, Germany; ^3^ Department of Otorhinolaryngology, Head and Neck Surgery, University of Ulm, Ulm, Germany; ^4^ Department of Hematology/Oncology, University of Leipzig, Leipzig, Germany; ^5^ Department of Internal Medicine III, University of Ulm, Ulm, Germany; ^6^ Division of Stem Cell Transplantation and Immunotherapy, 2nd Department of Medicine, University of Kiel, Kiel, Germany; ^7^ Department of Medicine III, Johannes Gutenberg-University Mainz, Mainz, Germany; ^8^ Department of Hematology/Oncology, Georg-August-University Göttingen, Göttingen, Germany; ^9^ Department of Stem Cell Transplantation, University Hospital Hamburg Eppendorf, Hamburg, Germany; ^10^ Department Internal Medicine I, Universitätsklinikum des Saarlandes, Homburg, Germany; ^11^ Department of Internal Medicine II, University Hospital Würzburg, Würzburg, Germany; ^12^ Medizinische Klinik 5, Klinikum Nürnberg, Paracelsus Medizinische Privatuniversität, Nürnberg, Germany; ^13^ Department of Medicine III, Hematology/Oncology/Palliative Care, Rostock University Medical Center, Rostock, Germany; ^14^ Division of Hematology and Oncology, Oldenburg Clinic, University of Oldenburg, Oldenburg, Germany; ^15^ 2nd Department of Internal Medicine, Oncology and Hematology, Robert Bosch Hospital Stuttgart, Stuttgart, Germany; ^16^ Department of Hematology/Oncology and Stem Cell Transplantation, Evangelisches Krankenhaus Essen-Werden, Essen, Germany; ^17^ Department of Hematology/Oncology, Klinikum Bremen-Mitte, Bremen, Germany; ^18^ Medizinische Klinik III, Universitäts Medizin Mannheim, Mannheim, Germany; ^19^ Medizinische Klinik III, Städtisches Klinikum Karlsruhe, Karlsruhe, Germany; ^20^ ZKRD - Zentrales Knochenmarkspender-Register für Deutschland, German National Bone Marrow Donor Registry, Ulm, Germany; ^21^ DRST – German Registry for Stem Cell Transplantation, Ulm, Germany

**Keywords:** stem cell transplantation, graft-versus-host-disease, HLA-DPB1, HLA-DPB1 expression, HLA-DPB1-permissiveness

## Abstract

T-cell epitope matching according to the TCE3 algorithm classifies HLA-DPB1 mismatches in permissive and non-permissive. This classification has been shown to be predictive for mortality and acute GvHD (aGvHD) events in large international cohorts. We retrospectively genotyped HLA-DPB1 in 3523 patients transplanted in Germany between 2000 and 2014 and in their unrelated donors using an Illumina amplicon-NGS based assay. Aim of the study was to evaluate DP-compatibility beyond the established TCE3 algorithm by assessing the combined effect of several DP-mismatch parameters on post-transplant outcome. We implemented an extended DP-mismatch assessment model where TCE3, DP allotype expression with respect to rs9277534, mismatch vector and number of mismatches were conjointly taken into consideration. In this model, non-permissive HLA-DPB1 mismatches showed significantly increased aGvHD risk if they were accompanied by two HLA-DPB1 mismatches in GvH direction (HR: 1.46) or one mismatched highly expressed patient allotype (HR: 1.53). As previously reported, non-permissive HLA-DPB1 mismatches associated with a significantly higher risk of aGvHD and non-relapse mortality (HR 1.36 and 1.21, respectively), which in turn translated into worse GvHD and relapse free survival (HR 1.13). Effects on GvL and GvHD appeared strongest in GvH-directed non-permissive mismatches. Our study results support the consideration of additional HLA-DPB1 mismatch parameters along with the established TCE3 matching algorithm for refinement of future donor selection. In particular, our findings suggest that DP non-permissiveness associated with two HLA-DPB1 mismatches or at least on highly expressed mismatched patient allotype should be avoided.

## Introduction

Allogeneic hematopoietic stem cell transplantation has become an established clinical treatment for various, otherwise often incurable diseases of the lympho-hematopoietic system. Improvements in treatment protocols as well as donor selection procedures have led to increasing numbers of patients undergoing hematopoietic stem cell transplantation (HSCT) (1). Although the first choice is usually an HLA-identical sibling, often such donors are not available and therefore unrelated donors are used (2). As the segregation of haplotypes in unrelated donors cannot be determined, only locus-wise matching is performed and depending on the frequency of the patient’s HLA-phenotype, sometimes HLA-differences have to be accepted (3). It has become apparent, that matching for the antigen recognition domain (ARD) for classical HLA-loci improves post-transplant mortality and morbidity (4). The minimal consensus on compatibility testing requires high resolution typing for HLA-A, -B, -C, and -DRB1. Many centers in Europe also include HLA-DQB1 compatibility in donor selection strategies. The relevance of HLA-DPB1 matching in unrelated stem cell transplantation has long remained undefined. This may be due to several characteristics that distinguish HLA-DPB1 antigens from other classical HLA-molecules. First, HLA-class II molecules are formed as heterodimers of an alpha and a beta chain; the ARD is formed by the alpha-1 and the beta-1 domain. Most polymorphisms are located within the beta-1 domain (exon 2 of the respective gene). These polymorphisms are almost evenly distributed across allotypes of classical HLA-molecules. In contrast, for HLA-DPB1, most of the polymorphisms are observed within six polymorphic regions throughout exon 2 of the HLA-DPB1 gene, resulting in several clusters with similar immunogenicity (5). This leads to significantly less diversity regarding T-cell epitopes. Second, the linkage disequilibrium between classical HLA-genes is very strong, particularly for HLA-B/C and HLA-DR/DQ genes, forming conserved haplotypes, which are frequently observed (6). However, the linkage between HLA-DPB1 and other classical HLA-genes is rather low because of a recombination hotspot between the HLA-DQ and HLA-DP genes, which in turn occasionally leads to HLA-DPB1 disparities among apparently HLA-identical sibling donors and far more often to HLA-DPB1 incompatibility between recipients and their otherwise fully HLA-matched unrelated donors (~80%) (7, 8). Third, the expression of HLA-DPB1 is similar to that of HLA-DRB3/4/5 and HLA-DQB1 antigens and lower as compared to the classical HLA-antigens HLA-A, -B, -C, and -DRB1 (9, 10). The former are therefore referred to as low expression loci (LEL) and the latter as high expression loci (HEL). Last, serological typing for HLA-DPB1 has always been much more difficult due to lack of suitable antisera. It has been shown that only two sets of dimorphic amino acid epitopes account for most of the serological reaction patterns observed, resulting in considerably less diversity compared to the other classical HLA-antigens (11).

Early studies had shown that the impact of HLA-DPB1 differences on the incidence of GvHD was balanced by a lower relapse rate and therefore did not translate into better survival outcomes (12). It was also recognized that HLA-DPB1 differences might have an additional detrimental effect on the presence of other mismatches. Later it was discovered using cytotoxicity assays that HLA-DPB1 alleles may be grouped according to their T-cell immunogenicity into three groups (13). This led to the T-cell epitope matching algorithm, which allows grouping of DP-mismatches between patient and donor in permissive and non-permissive and which has been shown to associate with clinical outcome in large retrospective cohorts (7, 14). Another proposed model relates to the expression levels of HLA-DPB1 mismatches, which is influenced by an SNP in the 3’-UTR of HLA-DPB1 alleles (rs9277534) (15). Aim of our study was to validate these models in an independent cohort and to explore, if the two models are possibly complementary.

## Patients and Methods

### Study Cohort

This study included patients transplanted for various hematological diseases with peripheral blood stem cells (PBSC) or bone marrow (BM) from an unrelated donor at German centers. The transplants were performed from 2000 to 2014. All searches were conducted by the search unit in Ulm. Only transplants with first allogeneic transplantation were included. Disease status at time of transplantation was classified according to the definitions used in the establishment of the EBMT risk score (16). Myeloablative conditioning (MAC) was classified according to the definitions for standard intensity conditioning regimens of the EBMT MED-AB manual Appendix III and published consensus suggestions (17). Less intense regimens were considered as reduced intensity (RIC). Most of the patients received in-vivo T-cell depletion with ATG or Campath. Standard of post-Tx immunosuppression was a cyclosporine based treatment approach in the vast majority of cases. Study design, collection of clinical data and ethics aspects are described in detail in the **Supplemental Material**.

### HLA-Typing

For all patients high resolution HLA-typing was available for the gene loci HLA-A, -B, -C, -DRB1 and -DQB1, defining all polymorphisms within the ARD – exons 2 and 3 for HLA-class I, and exon 2 for HLA-class II molecules (18). Non-expressed alleles were excluded according to NMDP confirmatory typing requirements. For HLA-DPB1 retrospective typing was applied based on an NGS-amplicon sequencing methodology using the Illumina (San Diego, CA, USA) platform. This in-house protocol was validated and CE-certified as IVD-reagent and is routinely used in stem cell donor typing. HLA-alleles are considered as matched if they show the same protein sequence within the ARD.

### Definitions

HLA-DPB1 TCE3 matching was performed according to the revised TCE3 matching procedure based on functional distance (19). DPB1 mismatches were classified as permissive and non-permissive. In some models for non-permissive mismatches mismatch directionality (i.e. GvH vs HvG) was considered. Prediction of SNP rs9277534 was based on HLA-DPB1 genotyping using imputed information as previously described (20). With respect to rs9277534, DPB1 mismatches were categorized into two surface expression groups (G allele as high and A allele as low expressed). In the combined DP mismatch model, TCE3, rs9277534, mismatch vector as well as number of mismatches were conjointly taken into consideration. Specifically, for the expression part only mismatched allotypes in GvH direction (the mismatched patient allotype) were considered ranging from matched to zero mismatches in GvH vector, one mismatch in GvH vector and “low-expressed” (rs9277534-A), one mismatch in GvH vector and “high expressed”(rs9277534-G) and both mismatched alleles irrespective of rs9277524 genotype. For the immunogenicity part the hierarchy with increasing risk was DP matched, DP permissive mismatched and DP non-permissive mismatched. As to the overall number of DP mismatches, this was calculated on the basis of GvH direction only.

Endpoints of interest were overall survival (OS), GvHD and relapse-free survival (GRFS), non-relapse mortality (NRM), aGvHD incidence and relapse incidence. OS was defined as time to death or last follow-up. GRFS was defined as time to aGvHD, relapse or death, whichever occurred first. NRM was defined as time to death from any cause except relapse. A relapse event was treated as competing risk. The endpoint aGvHD incidence was defined as time to first diagnosis of aGvHD (grades II-IV). An additional subanalysis for aGvHD (grades III-IV) was conducted. Death from any cause without prior aGvHD was considered as competing risk. Relapse incidence was defined as time to relapse and death from any cause without prior relapse was treated as competing risk. Patients alive and/or free from the event of interest were censored at last follow-up (21).

### Statistical Analysis

For descriptive statistics, the chi-squared test was used for categorial variables and the Mann-Whitney-U-Test for continuous variables. For survival analyses of the endpoints OS and GRFS Kaplan-Meier estimates were used and comparisons were performed with the log-rank test (22). For the endpoints NRM, aGvHD and relapse, cumulative incidence curves for competing risk data were generated and compared with the method of Gray (23). For multivariate analyses cause specific Cox models have been used, allowing for adjustment of time-dependent covariate effects in a piecewise constant manner (24). The breakpoints were chosen graphically (22). A center effect was adjusted. As this study represents a validation study of previous analyses, a significance level of 0.05 was considered sufficient for confirmation.

## Results

### High Prevalence of HLA-DPB1 Mismatches in 10/10 HSCT 9/10 HLA-Matched Hematopoietic Stem Cell Transplantation

The cohort consisted of 10/10 (n=2450, 69.5%) and 9/10 HLA (i.e. HLA-A, -B, -C, -DRB1, -DQB1) matched transplant pairs (n=1073, 30.5%). The distribution of diagnoses was similar in both groups, median age was slightly lower in the 9/10 matched transplants. Details regarding the cohort’s features are shown in **Table 1**. Median follow-up was 52 months.

**Table 1:** Patient characteristics.

	10/10 (%)	9/10 (%)	Total	P-Value
**N**	2450	1073	3523	n.a.
**Median age (range)**	54 (0–77)	52 (0–76)	53 (0–77)	0.014
**AML**	852 (34.8)	401 (37.4)	1253	0.466
**MDS**	375 (15.3)	161 (15.0)	536	
**NHL**	313 (12.8)	114 (10.6)	427	
**ALL**	273 (11.1)	131 (12.2)	404	
**Myeloma**	231 (9.4)	94 (8.8)	325	
**CLL**	135 (5.5)	54 (5.0)	189	
**Acute Leukemia**	120 (4.9)	45 (4.2)	165	
**other**	79 (3.2)	34 (3.2)	113	
**CML**	72 (2.9)	39 (3.6)	111	
**Early stage**	924 (37.7)	420 (39.1)	1344	0.531
**Intermediate stage**	837 (34.2)	346 (32.2)	1183	
**Advanced stage**	629 (25.7)	280 (26.1)	909	
**Unknown or n.a.**	60 (2.4)	27 (2.5)	87	
**KPS 80-100**	1,875 (76.5)	740 (69.0)	2615	0.119
**KPS <80**	116 (4.7)	60 (5.6)	176	
**Missing**	459 (18.7)	273 (25.4)	732	
**BM**	146 (6.0)	85 (7.9)	231	0.036
**PBSC**	2,304 (94.0)	987 (92.0)	3291	
**Missing**	0 (0)	1 (0.1)	1	
**MAC**	1,501 (61.3)	699 (65.1)	2200	**0.033**
**RIC**	948 (38.7)	374 (34.9)	1322	
**Missing**	1 (0)	0 (0)	1	
**ATG/Campath**	1,652 (67.4)	701 (65.3)	2353	0.400
**No ATG/Campath**	514 (21.0)	193 (18.0)	707	
**Missing**	284 (11.6)	179 (16.7)	463	
**Donor Age 18-30**	845 (34.5)	309 (28.8)	1154	**<0.001**
**Donor Age 31-45**	1,174 (47.9)	480 (44.7)	1654	
**Donor age 46-60**	363 (14.8)	211 (19.7)	574	
**Missing**	68 (2.8)	73 (6.8)	141	
**P-D CMV neg neg**	787 (32.1)	299 (27.9)	1086	**<0.001**
**P-D CMV neg pos**	205 (8.4)	111 (10.3)	316	
**P-D CMV pos neg**	569 (23.2)	306 (28.5)	875	
**P-D CMV pos pos**	760 (31.0)	294 (27.4)	1054	
**Missing**	129 (5.3)	63 (5.9)	192	
**P-D ABO match**	995 (40.6)	418 (39)	1413	0.421
**P-D ABO major**	551 (22.5)	249 (23.2)	800	
**P-D ABO bidir**	223 (9.1)	115 (10.7)	338	
**P-D ABO minor**	613 (25.0)	262 (24.4)	875	
**Missing**	68 (2.8)	29 (2.7)	97	

Acute Leukemia, undifferentiated, biphenotypic, secondary or unclassified; n.a., not applicable; KPS, Karnofsky performance score; BM, bone marrow; PBSC, peripheral blood stem cells; MAC, myeloablative conditioning; RIC, reduced intensity conditioning; P-D Patient-Donor; major, major incompatibility; bidir, bidirectional incompatibility; minor, minor incompatibility.

Retrospective genotyping of HLA-DPB1 locus in patients and their respective donors confirmed the high prevalence of HLA-DPB1 mismatches in both, 10/10 and 9/10 HLA-matched transplantations already reported elsewhere (7, 25, 26). Specifically, in the subgroup of 10/10 HLA-matched transplantations only 21.3% (n=521) were HLA-DP identical, while in the subgroup of 9/10 HLA-matched this fraction was 18.5% (n=198). Further categorization of DP mismatches as to permissiveness according to TCE3 revealed that in 37.9% (n=929) of 10/10 and in 34.9% (n=375) of 9/10 matched transplantations, respectively, the DP mismatch was permissive. For the remainder of the transplantations the HLA-DPB1 mismatches were non-permissive with even sub-distributions into the GvH and HvG vector. Almost half of the transplantations were single DP-mismatches, while 30% showed two DP differences. A double DP mismatch in GvH direction regardless of permissiveness was seen in about 23% of the cases. These data are summarized in **Table 2**. Additional multivariate analyses considering separately 10/10 and 9/10 HLA matched cases showed that the HLA-DP mismatch effect remained constant and uninfluenced by the presence of an additional HLA mismatch with the exception of relapse. The latter is analyzed in more detail right after. The data of these analyses are presented in detail in the **Supplemental Material** [**S5-S7, Supp3-Supp7(F)**].

**Table 2:** Results of HLA-DPB1 TCE3 matching.

	10/10 (%)	9/10 (%)	Total
**DP matched**	521 (21.3)	198 (18.5)	719
**DP permissive MM**	929 (37.9)	375 (34.9)	1304
**DP non-permissive GvH vector**	493 (20.1)	241 (22.5)	734
**DP non-permissive HvG vector**	507 (20.7)	259 (24.1)	766
**DP non-permissive MM total**	1,000 (40.8)	500 (46.6)	1,500
**DP 1 MM**	1,206 (49.2)	522 (48.6)	1,728
**DP 2 MM**	723 (29.5)	353 (32.9)	1,076
**DP non-permissive 2MM GvH vector**	377 (15.4)	196 (18.2)	573
**DP permissive 2MM GvH vector**	165 (6.7)	70 (6.5)	503

MM, Mismatch; GvH, Graft versus Host; HvG, Host versus Graft.

### Known Associations of Non-Permissive HLA-DPB1 Mismatches With Outcome Endpoints Confirmed

**Figure 1:**
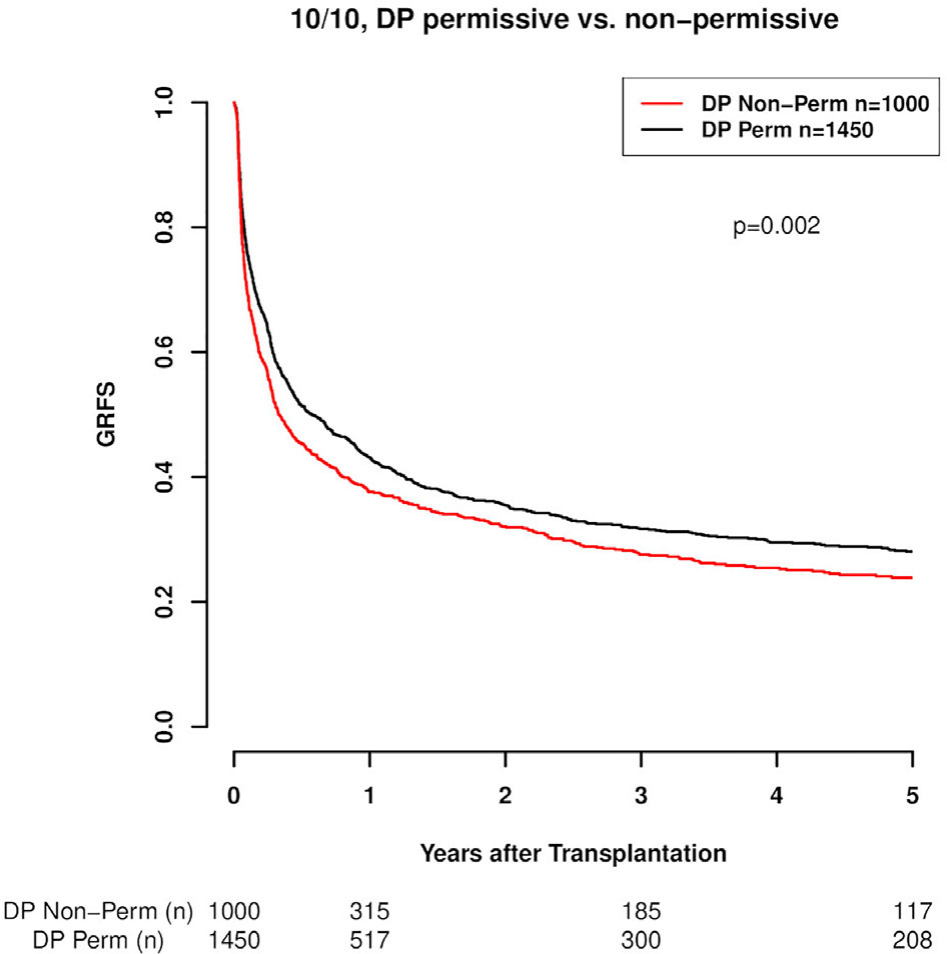
GRFS (Graft vs Host disease- and relapse-free survival; GvHD II-IV) in 10/10 HLA-matched cases with respect to DP mismatch permissiveness according to TCE3. DP Perm vs. DP Non-Perm cases, where DP Perm = DP matched or TCE3 permissively mismatched and DP Non-Perm = DP TCE3 non-permissively mismatched (p=0.002).

**Figure 2:**
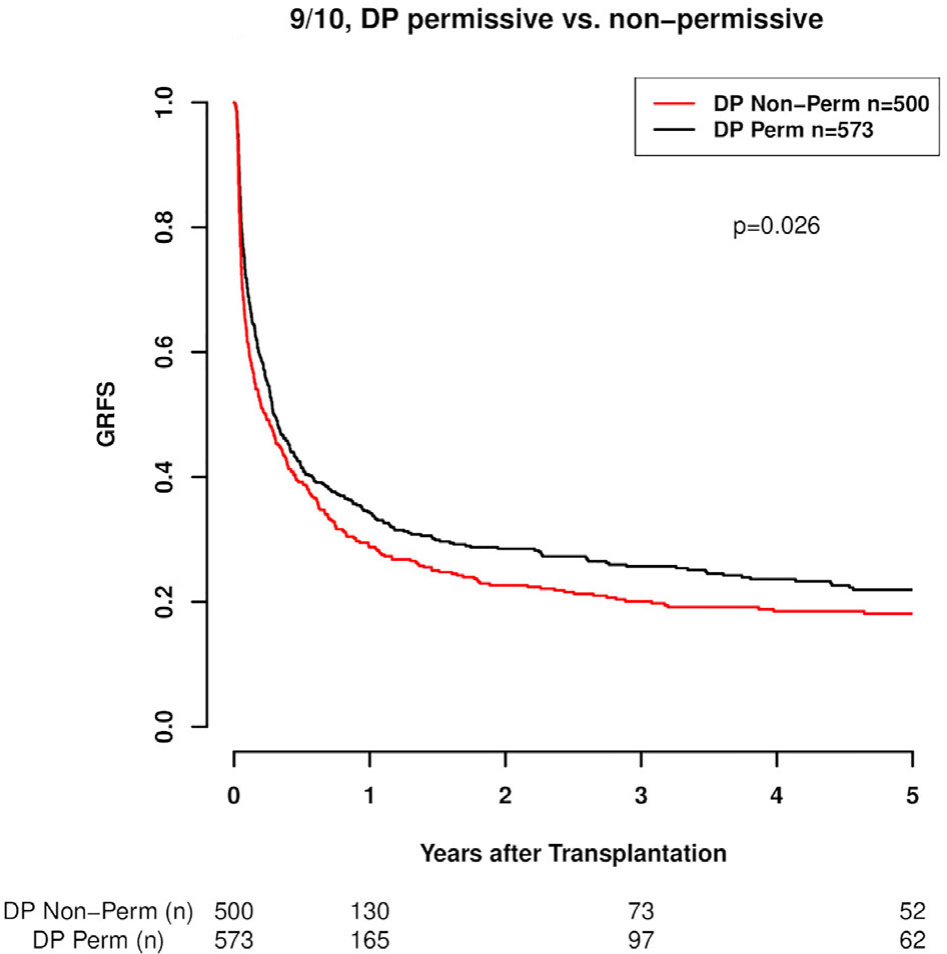
GRFS (Graft vs Host disease- and relapse-free survival; GvHD II-IV) in 9/10 HLA-matched cases with respect to DP mismatch permissiveness according to TCE3. DP Perm vs. DP Non-Perm cases, where DP Perm = DP matched or TCE3 permissively mismatched and DP Non-Perm = DP TCE3 non-permissively mismatched (p=0.026).

Regarding the effect of HLA-DPB1 mismatch on outcome endpoints, our results are in line with those previously reported. Specifically as to GRFS, 10/10 HLA-matched transplant pairs with HLA-DP non-permissive mismatches compared to HLA-DP matched or permissively mismatched cases exhibited a clearly higher composite risk of relapse, GvHD II-IV or death as presented in the GRFS outcome endpoint. These results are graphically depicted in **Figure 1** (p=0.002) and in more detail presented in **Table 3**. A similar result was seen in the subgroup of 9/10 matched transplantations where also patients with a DP matched or permissively mismatched donor showed significantly better GRFS (p=0.026), (**Figure 2**, **Table 3**). Multivariate analysis confirmed the results of the univariate models with non-permissive DP mismatches associating with significantly inferior GRFS (HR 1.16, CI 1.08–1.26, p<0.001, **Table 4**). No significant difference was observed between TCE3 permissively mismatched and fully DP matched transplantations with regard to this endpoint (HR 0.95, CI 0.86–1.06, p=0.401). Another GRFS analysis considering GvHD III-IV led to similar findings (**Table S2** in Supplemental material). It is of note that in the composite endpoint GRFS, the impact of TCE3 matching showed a time-dependent effect for non-permissive mismatches. This effect was only significant in the first 100 days after transplantation (non-permissive until d100: HR 1.23, CI 1.11-1.36, p<0.001 and non-permissive GvH direction until d100: HR 1.31, CI 1.16–1.48, p<0.001). Afterwards, non-permissive mismatches showed only a non-significantly increased risk of worse GRFS (**Table S1** in Supplemental Material). Time-dependent effects of other clinical covariates were also modeled and are shown in **Table S1** in Supplemental Material. These time-dependent covariables resemble effects that were explored and published previously (24, 27).

**Table 3:** Univariate analysis.

Univariate Analysis						
	10/10 HLA-matched HSCT			9/10 HLA-matched HSCT		
Endpoints	HLA-DP matched/permissive MM	HLA-DP non-permissive MM	p-value	HLA-DP matched/permissive MM	HLA-DP non-permissive MM	p-value
**GRFS (GvHD II-IV)**						
1 year	43.1% (40.5–45.9)	37.6% (34.6–40.9)		34.4% (30.5–38.7)	28.8% (24.9–33.2)	
3 year	31.7% (29.1–34.4)	27.6% (24.6–30.8)	**0.002**	25.7% (22.0–29.9)	20.1% (16.6–24.3)	**0.026**
5 year	28.0% (25.5–30.8)	23.9% (21.0–27.2)		21.9% (18.4–26.2)	18.1% (14.7–22.3)	
**GRFS (GvHD III**–**IV)**						
1 year	49.1% (46.5–51.9)	45.3% (42.2–48.7)		39.7% (35.8–44.1)	38.1% (35.8–44.1)	
3 year	34.4% (31.8–37.1)	31.9% (28.8–35.2)	**0.014**	29.6% (25.9–33.9)	25.2% (21.5–29.6)	0.191
5 year	29.0% (26.5–31.8)	27.5% (24.6–30.8)		23.3% (19.7–27.5)	21.8% (18.2–26.1)	
**Non-relapse mortality**						
1 year	20.9% (18.7–23.1)	25.6% (22.8–28.5)		27.0% (23.3–30.9)	33.1% (28.8–37.5)	
3 year	26.2% (23.8–28.7)	31.0% (27.9–34.1)	**0.010**	31.6% (27.6–35.7)	40.0% (35.3–44.6)	**0.013**
5 year	27.6% (25.1–30.2)	32.7% (29.6–36.0)		33.7% (29.5–38.0)	42.0% (37.2–46.7)	
**Relapse incidence**						
1 year	28.3% (25.9–30.8)	25.8% (23.0–28.7)		27.6% (23.5–31.8)	29.9% (26.0–33.9)	
3 year	38.5% (35.8–41.3)	34.3% (31.0–37.5)	**0.045**	36.2% (31.6–40.8)	37.3% (33.0–41.5)	0.201
5 year	41.9% (39.1–44.8)	37.0% (33.6–40.3)		38.4% (33.6–43.1)	41.8% (37.3–46.3)	
**aGvHD II**–**IV incidence**						
at day 100 after HSCT	22.9% (20.8–25.1)	29.4% (26.6–32.4)	**<0.001**	31.3% (27.5–35.2)	38.4% (34.1–42.6)	**0.010**

GRFS, GvHD and relapse free survival; GvHD, Graft versus Host Disease; MM, mismatch; HSCT, Hematopoietic Stem Cell Transplantation; HLA, Human Leukocyte Antigen. Statistical significance marked in bold.

**Table 4:** Multivariate analysis.

	OS		GRFS		NRM		Relapse			aGVHD II-IV		
	HR (CI)	P-value	HR (CI)	P-value	HR (CI)	P-value	HR (CI)		P-value	HR (CI)		P-value
**Patient Age**	1.02 (1.01–1.02)	**<0.001**	1.01 (1.01–1.01)*	**<0.001**	1.02 (1.02–1.03)	**<0.001**	1.01 (1.00–1.01)		**0.004**	–		
**Early stage disease**	1.00		1.00		1.00		1.00			–		
**Intermediate stage disease**	1.23 (1.08–1.40)	**0.002**	1.23 (1.11–1.37)	**<0.001**	1.02 (0.87–1.20)	0.816	1.66 (1.43–1.93)		**<0.001**	–		
**Advanced disease stage**	1.85 (1.63– 2.10)*	**<0.001**	1.53 (1.38–1.70)	**<0.001**	1.31 (1.13–1.53)*	**<0.001**	2.06 (1.77–2.38)*		**<0.001**	–		
**10/10 HLA**	1.00		1.00		1.00		–			1.00		
**9/10 HLA**	1.26 (1.14–1.40)	**<0.001**	1.26 (1.16–1.37)	**<0.001**	1.28 (1.12–1.46)	**<0.001**	–			1.41 (1.24–1.61)		**<0.001**
**Patient HLA-C KIR Ligand group C1x**	1.00		–		–		–			–		
**Patient HLA-C KIR Ligand group C2C2**	1.18 (1.04–1.35)	**0.012**	–		–		–			–		
**Donor age 18**–**30**	1.00		1.00		1.00		–			1.00		
**Donor age 31**–**45**	1.13 (1.01–1.27)	**0.029**	1.08 (0.99–1.19)	0.085	1.18 (1.02–1.37)	**0.028**	–			1.17 (1.01–1.35)		**0.037**
**Donor age 46**–**60**	1.25 (1.08–1.45)	**0.003**	1.12 (0.99–1.26)	0.067	1.48 (1.24–1.78)	**<0.001**	–			1.25 (1.04–1.51)		**0.017**
**P-D CMV neg-neg**	1.00		–		–		–			–		
**P-D CMV neg-pos**	1.13 (0.94–1.36)	0.190	–		–		–			–		
**P-D CMV pos neg**	1.14 (0.99–1.30)	0.061	–		–		–			–		
**P-D CMV pos pos**	1.09 (0.95–1.23)	0.210	–		–		–			–		
**RIC**	1.00		1.00		1.00		–			–		
**MAC**	1.23 (1.08–1.39)*	**0.001**	1.15 (1.04–1.27)	**0.007**	1.28 (1.11–1.47)*	**<0.001**	–			–		
**KPS 80**–**100**	1.00		1.00		–		1.00			–		
**KPS <80**	1.56 (1.26–1.93)	**<0.001**	1.37 (1.15–1.64)	**0.001**	–		1.38 (1.09–1.76)*		**0.007**	–		
**No in-vivo T-cell depletion**	1.00		1.00		–		1.00			1.00		
***In vivo* T-cell depletion**	0.84 (0.72–0.97)*	**0.015**	0.78 (0.69–0.88)*	**<0.001**	–		0.80 (0.69–0.92)		**<0.001**	0.67 (0.58–0.78)		**<0.001**
**Year of Tx 2000**–**2003**	–		1.00		1.00		1.00			1.00		
**Year of Tx 2004**–**2009**	–		0.56 (0.42–0.76)	**<0.001**	0.58 (0.40–0.84)	**0.005**	1.88 (1.10–3.24)		**0.022**	0.56 (0.37–0.84)		**0.005**
**Year of Tx 2010**–**2014**	–		0.57 (0.41–0.78)	**<0.001**	0.57 (0.39–0.84)	**0.004**	1.93 (1.11–3.34)		**0.020**	0.57 (0.37–0.86)		**0.008**
										
**TCE3 Permissive/DP matched**	1.00		1.00		1.00		1.00			1.00		
**TCE3 Non-permissive**	1.03 (0.94–1.14)	0.543	1.16 (1.08–1.26)*	**<0.001**	1.23 (1.09–1.39)	**<0.001**	0.90 (0.81–1.01)		0.094	1.33 (1.17–1.51)		**<0.001**
										
**DP matched**	1.00		1.00		1.00		1.00			1.00		
**TCE3 permissive**	0.91 (0.80–1.04)	0.179	0.95 (0.86–1.06)	0.401	0.97 (0.81–1.15)	0.693	0.91 (0.78–1.05)		0.198	1.04 (0.86–1.24)		0.698
**TCE3 Non-permissive**	0.97 (0.85–1.11)	0.662	1.13 (1.02–1.26)*	**0.025**	1.21 (1.02–1.42)	**0.029**	0.85 (0.74–0.99)		**0.033**	1.36 (1.15–1.61)		**<0.001**
										
**TCE3 Permissive/DP matched**	1.00		1.00		1.00		1.00			1.00		
**TCE3 Non-permissive GvH**	0.95 (0.84–1.08)	0.455	1.19 (1.08–1.31)*	**<0.001**	1.20 (1.03–1.41)	**0.014**	0.84 (0.73–0.97)		**0.018**	1.50 (1.29–1.75)		**<0.001**
**TCE3 Non-permissive HvG**	1.11 (0.98–1.24)	0.093	1.14 (1.03–1.26)	**0.001**	1.26 (1.09–1.46)	**0.002**	0.98 (0.85–1.12)		0.763	1.18 (1.01–1.38)		**0.037**

P-D, Patient-Donor; RIC, Reduced intensity conditioning,; MAC, Myeloablative conditioning; TCE3, T-cell epitope 3 matching; HR, Hazard ratio; CI, Confidence interval. Covariates showing time-dependent effects are labeled with an asterisk (*), -, not included in model. Statistical significance marked in bold.

**Figure 3:**
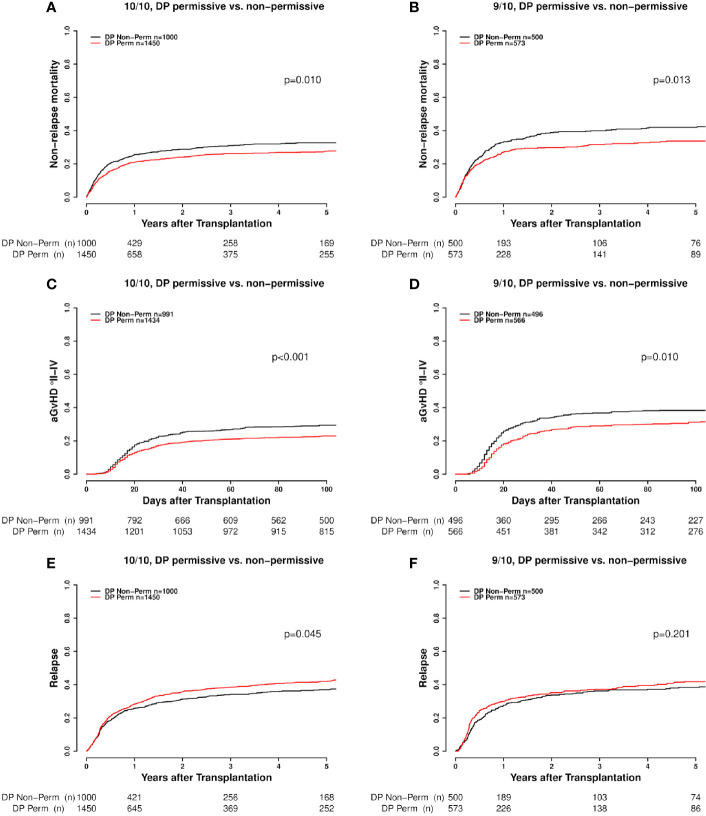
**(A–F)** Competing risks outcomes [non-relapse mortality (NRM), acute GvHD (aGvHD) and relapse] with respect to DP mismatch permissiveness according to TCE3 in 10/10 and 9/10 HLA-matched cases. DP Perm vs. DP Non-Perm cases, where DP Perm = DP matched or TCE3 permissively mismatched and DP Non-Perm = DP TCE3 non-permissively mismatched.

Non-relapse mortality was also significantly higher in DP non-permissive mismatched transplant pairs, both in the 10/10 HLA-matched (p=0.010, **Figure 3A**) and in the 9/10 HLA-matched group (p=0.013, **Figure 3B**). The results of the univariate analyses are shown in detail in **Table 3**. Again, the results of the univariate analysis were confirmed in the multivariate models, as non-permissive mismatches showed a significantly higher risk of NRM when compared to DP matched transplantations (HR 1.21. CI 1.02–1.42, p=0.029, **Table 4**). Permissive mismatches showed a risk similar to DP matched transplantations (HR 0.97, CI 0.81–1.15, p=0.693), as seen for GRFS. As expected the incidences of aGvHD were significantly higher in the HLA-DPB1 non-permissive mismatched groups in both univariate and multivariate models (**Tables 3** and **4**, **Figures 3C, D**). Analysis of the effect of HLA-DP MM on the incidence of chronic GvHD did not show significant results. The findings of this analysis are presented in the supplemental material (**Table S7**).As far as relapse incidence is concerned, both univariate and multivariate analyses clearly showed a significantly lower risk for HLA-DP non-permissive mismatches in otherwise 10/10 HLA-matched transplantations (p=0.045, **Figure 3E**). The respective results are shown in detail in **Tables 3** and **4**. Multivariate analysis of relapse incidence in patients with advanced disease stage confirmed the results of the whole cohort as to the effect of non-permissive DP mismatches (**S3** in **Supplemental Material**). Additional subanalyses comparing the DP-matched group separately vs. the TCE3 permissively and non-permissively mismatched group, respectively in 10/10 and 9/10 HLA matched transplantations revealed that DP matched cases exhibit a significantly higher risk of relapse compared to both, TCE3 permissively and non-permissively mismatched cases (HR 0.85, CI 0.73-0.99), p=0.038 for DP matched vs. TCE3 permissive mismatched; HR 0.81, CI 0.69-0.94), p=0.006 for DP matched vs. TCE3 non-permissively mismatched in 10/10 HLA transplantations). This was however, evident only in the 10/10 HLA matched setting, as the additional HLA mismatch appeared to completely abrogate that beneficial effect of DP mismatch (both permissive and non-permissive) vs. DP match as to lower relapse incidence. This was seen in both, multivariate and univariate models for relapse incidence. These data are presented in more detail in the supplemental material **(Supp7(E), Supp7(F), S5** and **S6**). Furthermore the impact of DP mismatches on OS was not statistically significant in either univariate (data not shown) or multivariate analyses (**Table 4**). Last, a significant impact of CMV matching status on transplantation outcome was seen neither in the 10/10 nor the 9/10 HLA-matched group (**Table S5** and **S6**).

### Mismatch Directionality Relevant Only in aGvHD and Relapse

Subanalysis of the vector of non-permissiveness against DP matched/permissively mismatched showed significantly higher risks for both GvH and HvG directed mismatches in the GRFS endpoint. (Non-permissive GvH: HR 1.19, 1.08–1.31, p<0.001 and non-permissive HvG: HR 1.14, 1.03–1.26, p=0.001). The detrimental effect of the non-permissive mismatches on NRM was again independent of the mismatch directionality as both, GvH and HvG vector non-permissive mismatches, associated with increased NRM risk (GvH: HR 1.20, CI 1.03–1.41, p=0.014; HvG: HR 1.26, CI 1.09–1.46, p=0.002). Contrary to the previous endpoint analyses, the mismatch vector appeared to indeed play a role in aGvHD incidence, as the higher risk observed was mostly driven by non-permissive mismatches in GvH direction (HR 1.50, CI 1.29–1.75, p<0.001). In line with the results for aGvHD, the effect of non-permissive mismatches on relapse incidence appeared to be mainly driven by the GvH vector (GvH direction: HR 0.84, CI 0.73–0.97, p=0.018; HvG direction: HR 0.98, CI 0.86–1.12, p=.0763; **Table 4**). It is of note that this vector effect was not seen in the subanalysis of patients with advanced disease, as no differences were seen between GvH and HvG vectors (**Table S3** in Supplemental Material).

### Effect of Non-Permissive HLA-DPB1 Mismatch Aggravated by Increasing Number of DP Mismatches and High Expression Level of Mismatched Allotype in GvH Vector

In the combined DP mismatch (TCE3-rs9277534) model, an interesting finding was that apart from TCE3 permissiveness also the overall number of DP mismatches as well as the anticipated expression level of the mismatched allotype with a GvH vector contributed to the overall mismatch effect. Specifically, a significantly higher risk of GRFS, NRM and aGvHD was found for DP non-permissive mismatches with two overall DP-allele-mismatches in GvH direction (**Table 5**). For aGvHD incidence, also DP non-permissive mismatches with one high expressed mismatched patient allotype (rs9277534-G) showed significantly higher risk estimates (**Table 5**). Conversely, with respect to relapse incidence, these categories associated with significantly lower risk as shown in **Table 5**. The enhancement of non-permissive DP effect on the aforementioned endpoints becomes clear after comparison of the respective hazard risks for non-permissive DP mismatches overall and for non-permissive DP mismatches with highly expressed patient mismatched allotype or double mismatch in GvH direction as presented in **Tables 4** and **5**. Although statistical significance was not reached in the subgroup of two overall permissive mismatches in GvH direction, a clear trend was seen at least for aGvHD (**Table 5**).

**Table 5:** Combined DP-mismatch model.

	OS		GRFS		NRM		Relapse		aGVHD II-IV	
	HR (CI)	P-value	HR (CI)	P-value	HR (CI)	P-value	HR (CI)	P-value	HR (CI)	P-value
**DP matched, N=719**	1.00		1.00		1.00		1.00		1.00	
**DP Permissive MM, 0MM GvH, N=6**	0.80 (0.63–1.01)	0.060	0.78 (0.29–2.10)	0.622	0.60 (0.08–4.26)	0.605	0.69 (0.17–2.78)	0.602	0.72 (0.10–5.14)	0.742
**DP Permissive MM, 1MM GvH (A), N=742**	0.49 (0.12–1.97)	0.312	0.91 (0.80–1.03)	0.119	0.92 (0.75–1.13)	0.433	0.88 (0.74–1.04)	0.122	0.94 (0.76–1.16)	0.551
**DP Permissive MM, 1MM GvH (G), N=321**	0.88 (0.76–1.03)	0.105	0.98 (0.84–1.16)	0.803	0.99 (0.77–1.27)	0.949	0.94 (0.77–1.16)	0.592	1.08 (0.83–1.40)	0.561
**DP Permissive MM, 2MM GvH, N=235**	0.92 (0.76–1.11)	0.360	1.17 (0.94–1.33)	0.215	1.10 (0.83–1.44)	0.515	0.97 (0.77–1.23)	0.803	1.30 (0.98–1.71)	0.065
**DP Non-Permissive MM, 0MM GvH, N=125**	1.02 (0.83–1.26)	0.825	1.01 (0.81–1.27)	0.916	1.09 (0.76–1.54)	0.648	1.14 (0.85–1.54)	0.371	0.93 (0.63–1.38)	0.721
**DP Non-Permissive MM, 1MM GvH (A), N=365**	1.07 (0.82–1.39)	0.641	1.05 (0.90–1.21)	0.563	1.08 (0.86–1.38)	0.494	1.02 (0.84–1.25)	0.831	1.15 (0.90–1.46)	0.264
**DP Non-Permissive MM, 1MM GvH (G), N=437**	0.93 (0.78–1.11)	0.428	1.13 (0.98–1.30)	0.094	1.16 (0.93–1.45)	0.187	0.76 (0.62–0.94)	**0.010**	1.53 (1.23–1.91)	**<0.001**
**DP Non-Permissive MM, 2MM GvH, N=573**	0.92 (0.77–1.09)	0.341	1.22 (1.07–1.39)	**0.002**	1.37 (1.13–1.67)	**0.002**	0.76 (0.63–0.92)	**0.004**	1.46 (1.19–1.79)	**<0.001**

MM, Mismatch; 0MM GvH, mismatched Allotype in GvH vector; 1MM GvH (A), 1 mismatched Allotype in GvH vector (rs9277534 A); 1MM GvH (G), 1 mismatched Allotype in GvH vector (rs9277534 G); 2MM, both DP alleles mismatched. Statistical significance marked in bold. Statistical trend is underlined.

## Discussion

Several factors seem to contribute to the alloreactivity is induced by HLA-DPB1 differences. These are linked to the intrinsic immunogenicity on account of T-cell epitopes, the numbers and vectors of mismatches as well as the expression level of the mismatched allele. HLA-DPB1 mismatching represents therefore a multilevel variable and any individual model represents only a simplification of the true biological relationship. In this retrospective study we sought to conjointly assess the effect of the aforementioned factors with the aim to offer a more unified approach as to DP mismatch evaluation for donor selection. Through our analysis we were able to confirm previously described associations, while we also showed that consideration of additional factors might be meaningful for histocompatibility assessment and for improving predictiveness.

Regarding the prevalence of HLA-DPB1 mismatches in 10/10 as well as 9/10 HLA-matched HSCTs, no differences were observed between our findings and those seen in other studies (7, 25, 26). The same applies for distribution of non-permissive mismatches in GvH and HvG direction, which was balanced in the respective immunogenicity models (28). HLA-DP matched and permissively mismatched transplants have been grouped together for this analysis, as the broadly used TCE algorithm tool makes no distinction between these two groups for which we only observed a difference in the relapse analysis as already mentioned before.

In line with previously reported associations of HLA-DPB1 non-permissive mismatches with outcome endpoints, we also observed a clearly higher risk of aGvHD and NRM (7, 14). Although the risk of relapse was significantly lower in the non-permissive mismatch group, the composite GRFS endpoint was overall inferior compared to the matched/permissively mismatched ones, most probably due to the detrimental effect of non-permissive HLA-DPB1 mismatches on aGvHD incidence. The higher induced T-cell alloreactivity most likely accounts for this effect, as this has been previously shown by in-vitro testing (5, 13). Interestingly, with respect to OS, the two opposite effects of NRM and relapse appear to have mutually eliminated one another, as no significant differences were observed with respect to DP mismatch permissiveness. Closer look into the death cause analysis in DP non-permissively mismatched and matched/permissively mismatched cases may explain the aforementioned observation on OS (death cause analysis results are presented in detail in the **Supplemental Material** section). Although non-permissive HLA-DPB1 mismatches significantly increased the risk of aGvHD, this didn’t translate into higher mortality. This doesn’t seem to be the case for relapse, where matched or permissively mismatched cases showed a markedly higher mortality related to relapse (42.6%) compared to non-permissively mismatched cases (33.8%), (data shown in Supplemental Material, **Table S4**). The additional HLA mismatch this time did not seem to impact the DP match effect as similar relapse-related death rates were observed for the DP compatibility groups analyzed in both, 10/10 and 9/10 HLA settings (data not shown). One additional factor is the time dependence of DP mismatch effect on GRFS, as from day 100 post HSCT it ceased to be significant (27).

Multivariate Models were checked for interaction between HLA-DPB1 and other classical HLA-mismatches by forming an interaction term, which showed not statistical significance. This is also shown in the separate analysis of 10/10 HLA and 9/10 HLA matched cases, where the respective effect of HLA-DPB1 mismatch did not appear to be influenced by the prevalence of an additional HLA mismatch with the exception of relapse as already mentioned previously. This implies that HLA-DP mismatches confer their effect on outcome rather independently from additional HLA-mismatches. As far as mismatch directionality is concerned, our analysis revealed that non-permissive mismatches in GvH direction mainly drove the overall effect of higher aGvHD but also lower relapse risk when compared to non-permissive mismatches in HvG direction. The fact that no such effect was observed in NRM suggests that DPB1-mismatch-induced morbidity is not only restricted to aGvHD but may also affect other pathophysiological pathways such as conditioning associated toxicity or infections early after hematopoietic stem cell transplantation (29). A mechanism of interaction may be the upregulation of HLA-class II molecules during viral infection possibly aggravating the impact of DPB1 mismatches in such cases (30). It’s also possible that this effect may be influenced by ATG/Campath treatment as well as post-transplant immunosuppression. This is supported by the fact that a similar effect was also seen in a cohort from the MD Anderson Cancer center (31) but not in a multicenter cohort of patients where the transplant was facilitated by the NMDP (27). As to the mismatch directionality effect on relapse, it could be immunobiologically underpinned by the notion that highly immunogenic patient mismatched HLA-DPB1 probably stimulates donor T-cells resulting in a better GvL effect. Similar observations have also been reported elsewhere (28, 32). Interestingly, this effect was not evident in the advanced-disease-stage patient group. This might be attributed, however, to weakened statistical power on account of multiple combinations.

In our analysis we explored the impact of HLA-DPB1 mismatches on GRFS, a composite endpoint now increasingly used for assessing the success of HSCT, as it simultaneously measures the proportion of patients free from disease and GvHD (33). We considered two degree levels for aGvHD, II-IV and III-IV. No marked differences were observed between the two subanalyses. As GRFS is a combined endpoint summarizing three events (occurrences of aGvHD, relapse or death), different effects are measured together. Perhaps the most interesting finding of this analysis was the absence of vector effect, although the latter was evident in aGvHD and relapse. An explanation for that could be the opposite effect of GvH directed non-permissive DP mismatches on these two endpoints resulting in an overall dampened and statistically insignificant effect.

Aim of this study was to conjointly assess different DP mismatch alloreactivity predictive models so that an extended predictive model can be proposed. To this end we included in our analysis, along with the TCE3 algorithm, the HLA-DPB1 expression model as proposed by Petersdorf et al. (14). Assignment of the rs9277534-G polymorphism was done by inference based on linkage disequilibrium data. A recent study showed that such an approach could be highly accurate (20). The G allele expression within the mismatched recipient allotype was associated with higher incidence of aGvHD suggesting a dose effect of the mismatched HLA-allotype (15). Such an association has also been reported for HLA-C differences (34). A shortcoming of the HLA-DPB1 expression model approach by Petersdorf et al. is that it was only applied to single mismatched HLA-DPB1 cases with no data available as to the effect of double mismatched HLA-DPB1 cases in GvH direction, which do however occur with a frequency of around 23%. In the combined DP mismatch model we aimed at combining the TCE3 immunogenicity- with the HLA-DPB1 expression-model taking also into consideration the mismatch directionality as well as the overall number of DP mismatches with GvH vector. This way we formed a hierarchy out of all implicated factors. The most important observation from this combined analysis consists in that HLA-DP non-permissive mismatch effect appears to be aggravated by the prevalence of two overall DP mismatches in the GvH direction as well as by an anticipated higher expressed patient mismatched allotype. The impact of two DP mismatches in GvH direction on non-permissive mismatch effect appears to be stronger as it significantly enhances the effect on GRFS, NRM, aGvHD and relapse. This observation is clinically relevant considering that about 16% of HSCTs are expected to have a non-permissive HLA-DP mismatch with two overall DP mismatches in GvH direction. The expected increased surface expression of the patient non-permissively mismatched allotype, on the other hand, appears to be significantly evident only for aGvHD and relapse. In summary these findings suggest that the combination of non-permissive DP mismatches with 2 DP-allele-mismatches as well as of non-permissive mismatches with a highly expressed mismatched patient allotype should be avoided. A recent study of Petersdorf et al. suggested that the overall number of mismatches is mainly relevant in HLA-mismatched transplantations whereas the expression level of the mismatched allotype is important in fully HLA-matched cases (35). Due to smaller cohort size and therefore compromised statistical power, we have not been able to confirm these findings in our study, as 10/10 and 9/10 HLA matched cases were assessed together in our combined DP-mismatch model. All other combinations including non-permissive mismatches with no mismatch in the GvH direction or a single low expressed mismatched allotype seem to be tolerable. This analysis is not yet conclusive as to whether double permissive DP-allele-mismatches in GvH direction should also be avoided or not, although a clear trend was also seen in this group. It is of note, however that this subgroup corresponded to only 6.7% of all included cases. Our study results indicate that although the immunogenicity model and the expression model confer distinctive effects on outcome due to different underlying mechanisms, they may be combined for refined donor selection strategies. Nevertheless, due to the many different possible combinations more data are needed and larger studies are warranted before final conclusions are drawn.

Limitations of our analysis are the small sample size in some sub-analyses particularly in the combined DP mismatch model. Missing data has also been a substantial problem for CMV status and blood group as well as for date of development of acute and chronic GvHD in the EBMT promise registry database although in direct collaboration with the transplant centers we were able to collect a substantial proportion of these missing data. Still missing data in the final analysis showed a completely random pattern, indicating no data collection bias. Furthermore, our cohort represents patients transplanted in Germany and shows a large proportion of patients treated with ATG as part of the conditioning treatment as well as a low proportion of patients treated with mTOR inhibitor based immunosuppression, which may limit comparability with other cohorts showing different features.

In conclusion, our study confirms the previously reported detrimental effect of non-permissive HLA-DPB1 mismatches according to the TCE3 model in a large cohort of patients having been treated with unrelated HSCT in German transplant centers between 2000 and 2014. This effect was similarly present in 10/10 and 9/10 HLA- matched transplantations. The results of our combined assessment of distinct DP mismatch alloreactivity models indicate that the effect mediated by rs9277534 may be independent from the immunogenicity model underlying the TCE3 model. Furthermore, an additional dose effect of mismatched HLA-DPB1 allotypes in GvH direction is implied, at least for aGvHD and relapse incidence. The aforementioned findings support an extension of the TCE3 model for refined donor selection avoiding the putatively detrimental combinations of non-permissive DP mismatches with overall 2 DP mismatches as well as with a high expressed mismatched patient allotype (rs9277534-G). Larger future studies are anticipated to offer a clearer insight into the multifaceted immunogenicity features of HLA-DPB1 mismatches addressed in this study.

## Data Availability Statement

The data analyzed in this study are subject to the following licenses/restrictions: If required, the data can be reanalyzed by other groups within our premises. Requests to access these datasets should be directed to joannis.mytilineos@zkrd.de.

## Ethics Statement

The studies involving human participants were reviewed and approved by the ethical committee of the University of Ulm. The patients/participants provided their written informed consent to participate in this study.

## Author Contributions

DF, DM, CT, JM, and HS are principal investigators. They designed the study, performed data analysis/interpretation, and wrote the manuscript. DM and CT as well as DF and JM contributed equally. MH, CM, SaF, CN, and CT contributed to the data analysis and in writing of the manuscript. CN, UP, DB, MG, ED, GW, NK, NM, HE, KE, SeF, JC, MK, MD, BH, SK, and MR contributed the patients, reviewed the data, and edited the manuscript. All authors contributed to the article and approved the submitted version.

## Funding

This work was supported by the Wilhelm Sander-Stiftung (Grant No 2018.092.1).

## Conflict of Interest

The authors declare that the research was conducted in the absence of any commercial or financial relationships that could be construed as a potential conflict of interest.
