# Supplementary material for: The Human Leukocyte Antigen-DPB1 Degree of Compatibility Is Determined by Its Expression Level and Mismatch Permissiveness: A German Multicenter Analysis
Source: Front Immunol. 2021 Jan 25;11:614976. doi: 10.3389/fimmu.2020.614976 (PMC7868530; doi:10.3389/fimmu.2020.614976)
Supplement: Supplementary file 1 [file DataSheet_1.pdf]

Supplemental Material

Study design, clinical data and ethics:

This study represents a retrospective analysis of the impact of HLA-DPB1 mismatches on the outcome of HSCT. HLA-typing for HLA-A, -B, -C, -DRB1 and -DQB1 was conducted upfront in the context of unrelated donor search, which was performed at the tissue typing laboratory of the search unit Ulm, where all donor searches were conducted. HLA-DPB1 typing was performed retrospectively. Individual treatment decisions were made by the transplanting centers, which also performed the follow-up. This information was recorded in the EBMT ProMISe database, which were made available for this study via the German registry for stem cell transplantation (DRST). Data was recorded at day 0, day 100 and every year thereafter. Missing data for ABO, CMV and date of aGvHD in the DRST file was requested directly from the centers. All patients and donors provided consent for HLA-typing. All patients provided consent for analysis of the clinical data for scientific purposes. The study was approved by the ethical committee of the University of Ulm. The procedures followed were in accordance with the Helsinki Declaration. We agree with the policy regarding deposition of publication-related data and sharing of reagents. The paper reports primary data. The manuscript has not been drafted and written by a ghost writer.

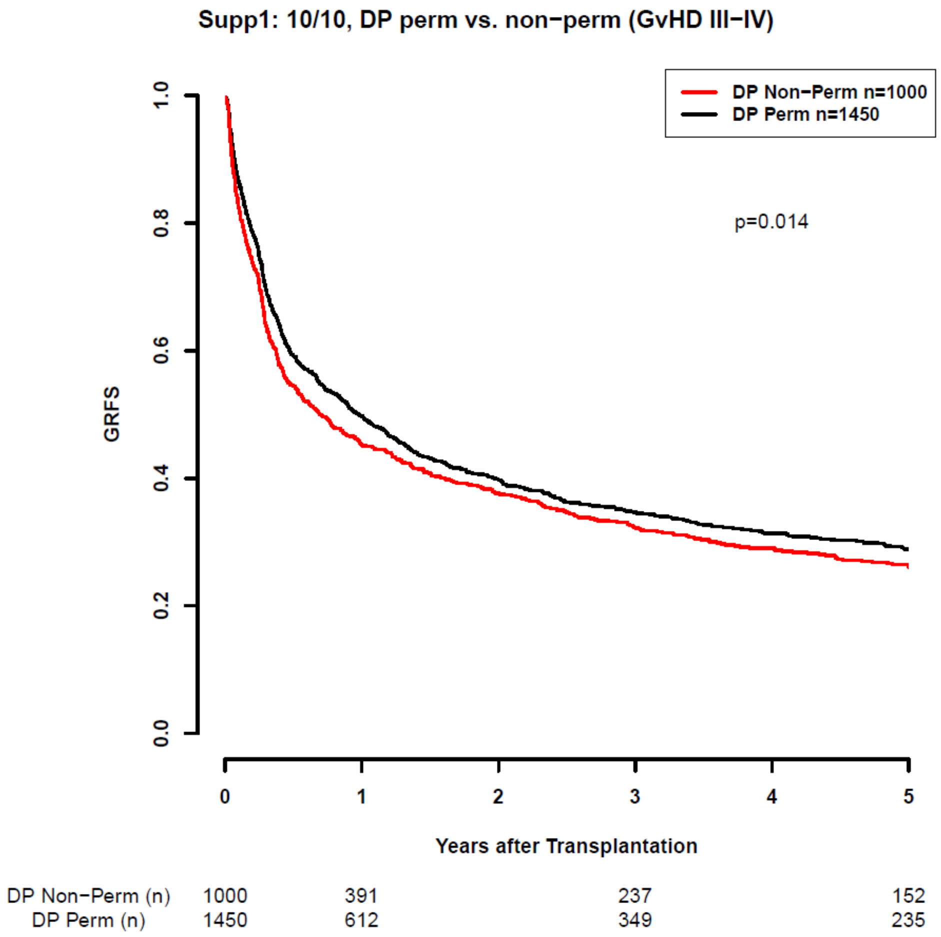

Supp1: GRFS in 10/10 HLA matched cases with respect to DP mismatch permissiveness according to TCE3. DP Perm vs. DP Non-Perm cases, where DP Perm = DP matched or TCE3 permissively mismatched and DP Non-Perm = DP TCE3 non-permissively mismatched (p=0.014). Graft vs Host disease- and relapse-free survival (GvHD III-IV)

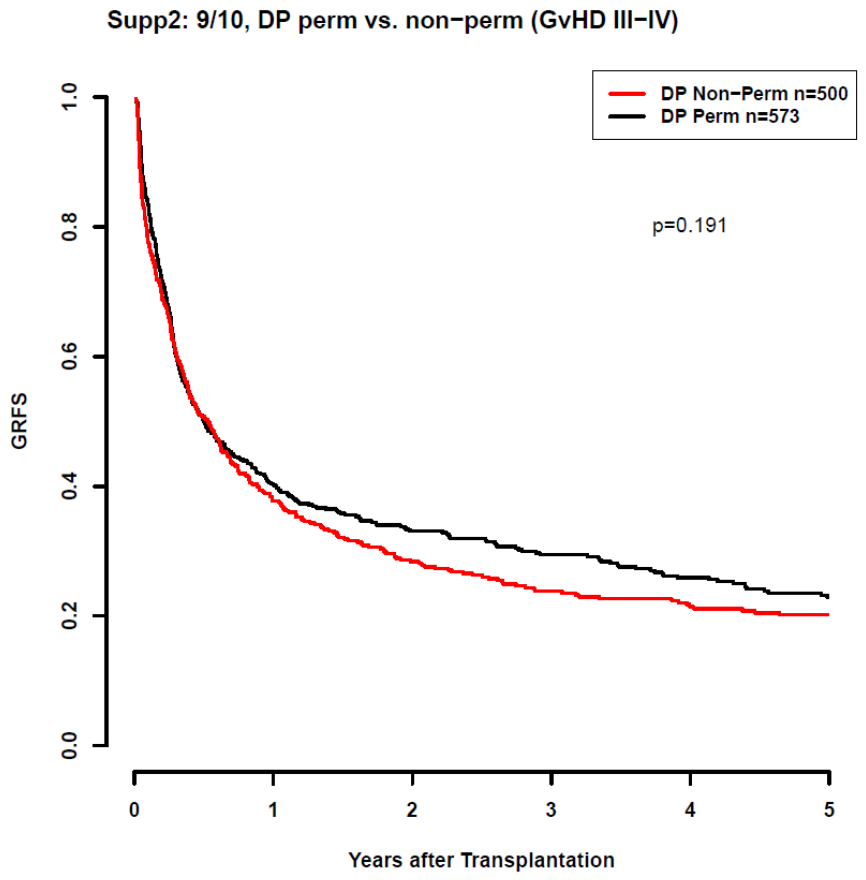

	0	1	3	5
DP Non−Perm (n)	500	174	95	68
DP Perm (n)	573	200	121	75

Supp2: GRFS in 9/10 HLA matched cases with respect to DP mismatch permissiveness according to TCE3. DP Perm vs. DP Non-Perm cases, where DP Perm = DP matched or TCE3 permissively mismatched and DP Non-Perm = DP TCE3 non-permissively mismatched (p=0.191). Graft vs Host disease- and relapse-free survival (GvHD III-IV)

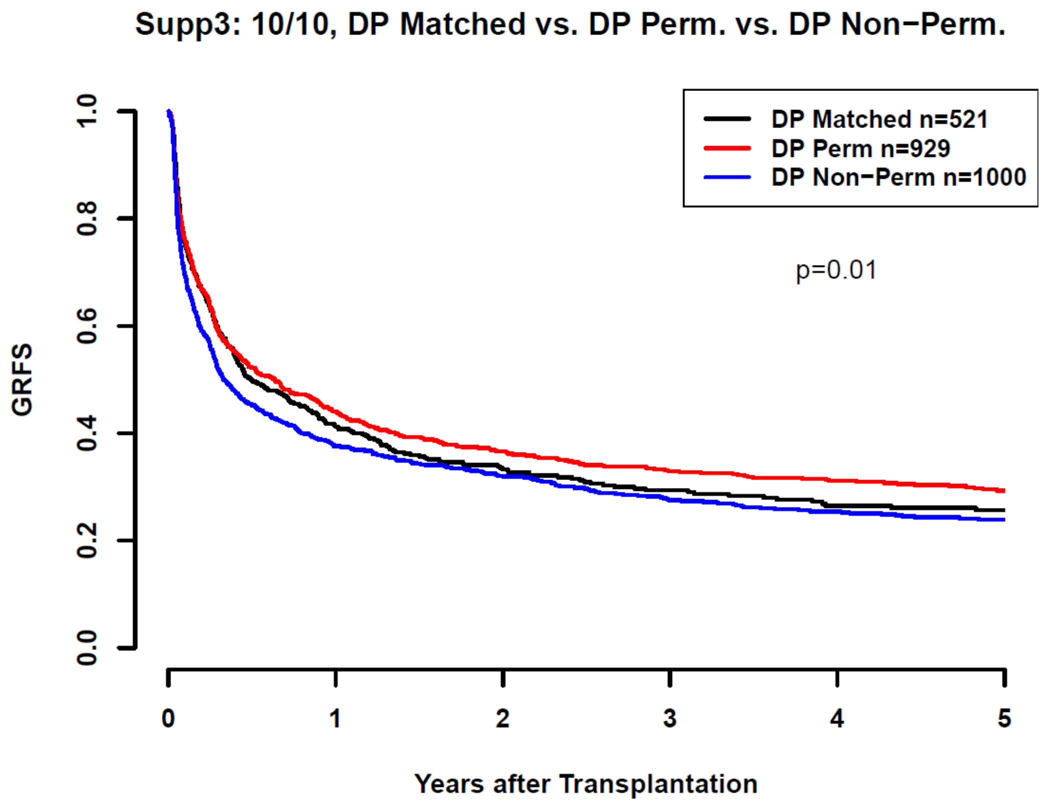

DP Matched (n)	521	167	94	59
DP perm. (n)	929	315	185	117
DP non−perm. (n)	1000	351	207	149

Supp3: GRFS in 10/10 HLA matched cases with respect to DP mismatch permissiveness according to TCE3. DP matched vs. DP Perm vs. DP Non-Perm cases, where DP Perm = TCE3 permissively mismatched and DP Non-Perm = DP TCE3 non-permissively mismatched (p=0.01). Graft vs Host disease- and relapse-free survival (GvHD II-IV)

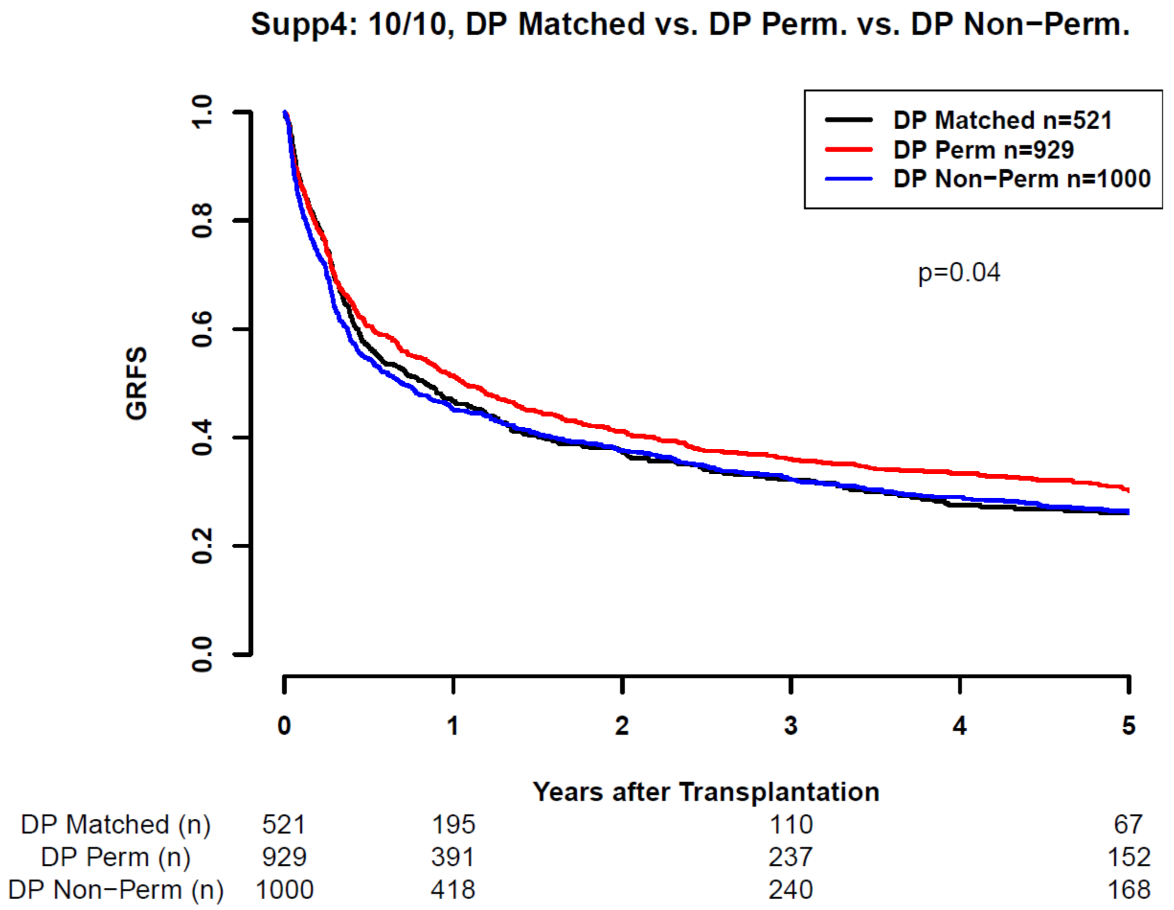

Supp4: GRFS in 10/10 HLA matched cases with respect to DP mismatch permissiveness according to TCE3. DP matched vs. DP Perm vs. DP Non-Perm cases, where DP Perm = TCE3 permissively mismatched and DP Non-Perm = DP TCE3 non-permissively mismatched (p=0.04). Graft vs Host disease- and relapse-free survival (GvHD III-IV)

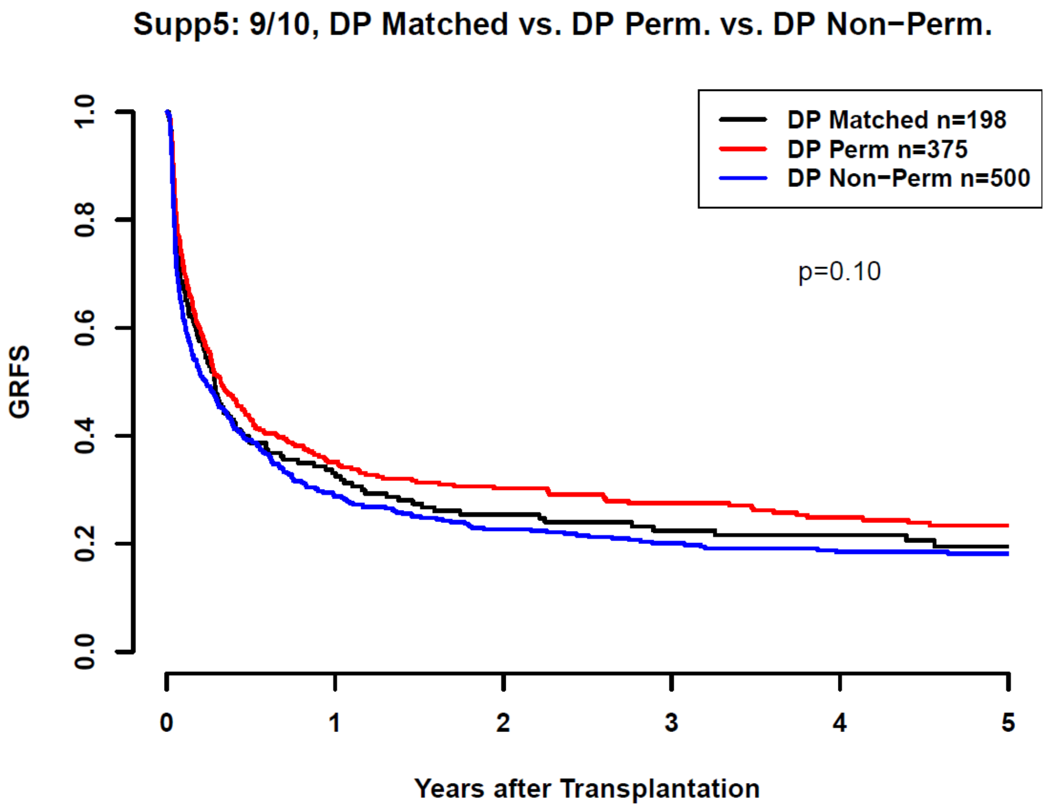

DP Matched (n)	198	54	29	18
DP Perm (n)	375	130	73	52
DP Non−Perm (n)	500	111	69	45

Supp5: GRFS in 9/10 HLA matched cases with respect to DP mismatch permissiveness according to TCE3. DP matched vs. DP Perm vs. DP Non-Perm cases, where DP Perm = TCE3 permissively mismatched and DP Non-Perm = DP TCE3 non-permissively mismatched (p=0.10). Graft vs Host disease- and relapse-free survival (GvHD II-IV)

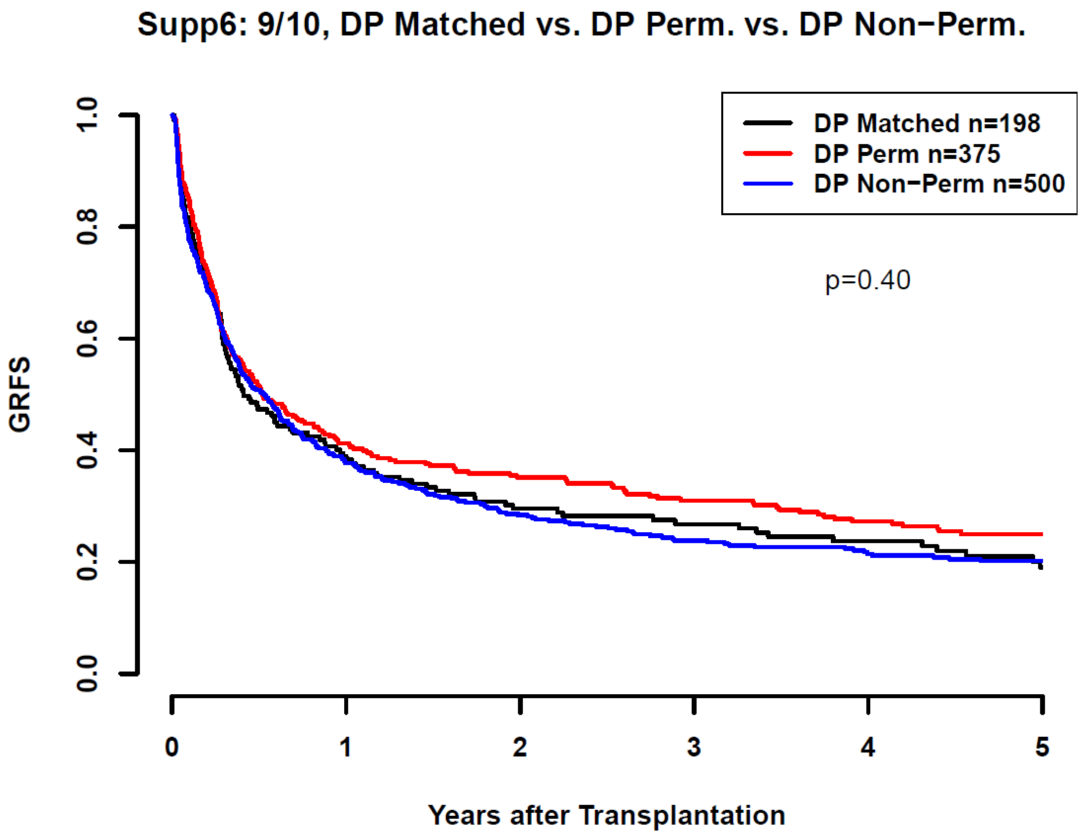

	0	1	3	5
DP Matched (n)	198	67	39	22
DP Perm (n)	375	174	95	68
DP Non−Perm (n)	500	133	83	54

Supp6: GRFS in 9/10 HLA matched cases with respect to DP mismatch permissiveness according to TCE3. DP matched vs. DP Perm vs. DP Non-Perm cases, where DP Perm = TCE3 permissively mismatched and DP Non-Perm = DP TCE3 non-permissively mismatched (p=0.40). Graft vs Host disease- and relapse-free survival (GvHD III-IV)

Supp7 (A): 10/10, DP Matched vs. DP Perm. vs. DP Non−Perm.

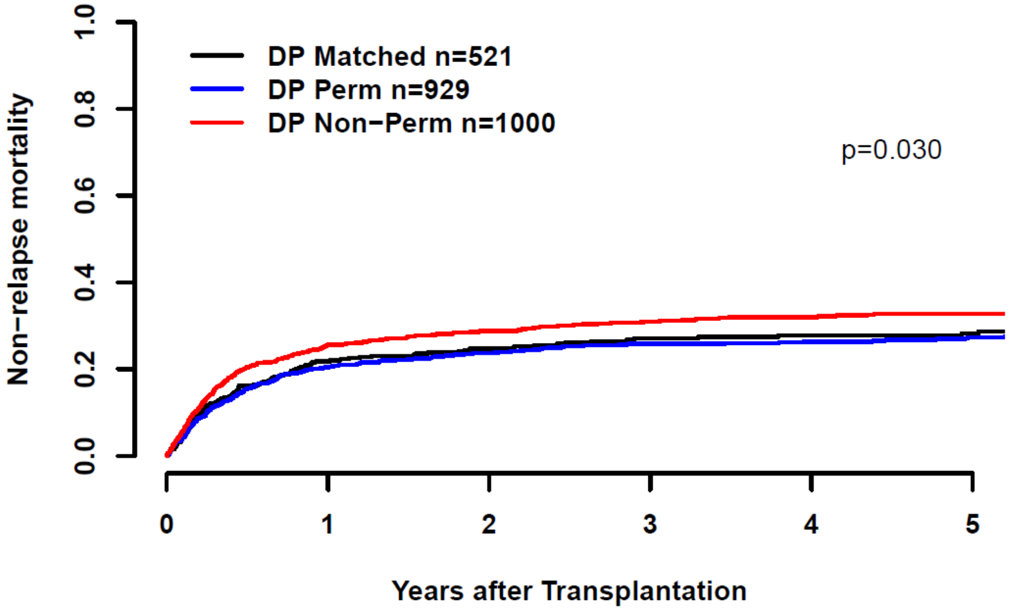

DP Matched (n)	521	207	118	73
DP Perm (n)	929	451	258	182
DP Non−Perm (n)	1000	429	258	169

Supp7(A): NRM in 10/10 HLA matched cases with respect to DP mismatch permissiveness according to TCE3. DP matched vs. DP Perm vs. DP Non-Perm cases, where DP Perm = TCE3 permissively mismatched and DP Non-Perm = DP TCE3 non-permissively mismatched (p=0.03).

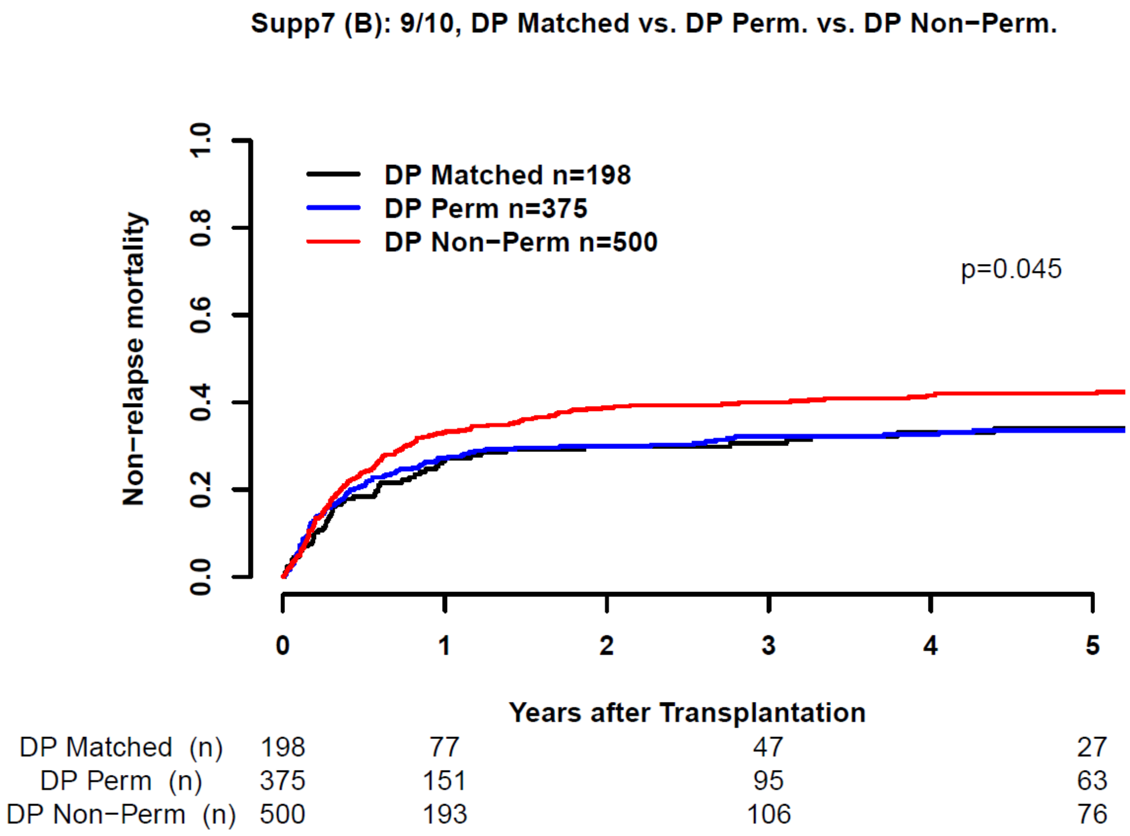

Supp7(B): NRM in 9/10 HLA matched cases with respect to DP mismatch permissiveness according to TCE3. DP matched vs. DP Perm vs. DP Non-Perm cases, where DP Perm = TCE3 permissively mismatched and DP Non-Perm = DP TCE3 non-permissively mismatched (p=0.045).

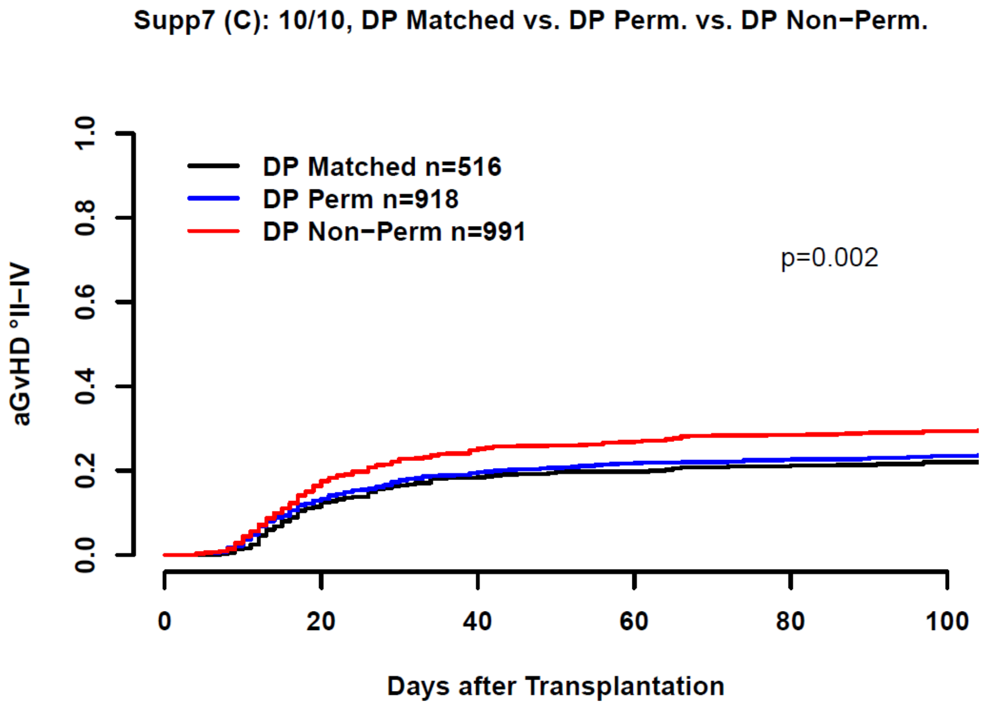

DP Matched (n)	516	438	374	349	324	290
DP Perm (n)	918	763	679	623	591	525
DP Non−Perm (n)	991	792	666	609	562	500

Supp7(C): aGvHD II-IV in 10/10 HLA matched cases with respect to DP mismatch permissiveness according to TCE3. DP matched vs. DP Perm vs. DP Non-Perm cases, where DP Perm = TCE3 permissively mismatched and DP Non-Perm = DP TCE3 non-permissively mismatched (p=0.002).

Supp7 (D): 9/10, DP Matched vs. DP Perm. vs. DP Non−Perm.

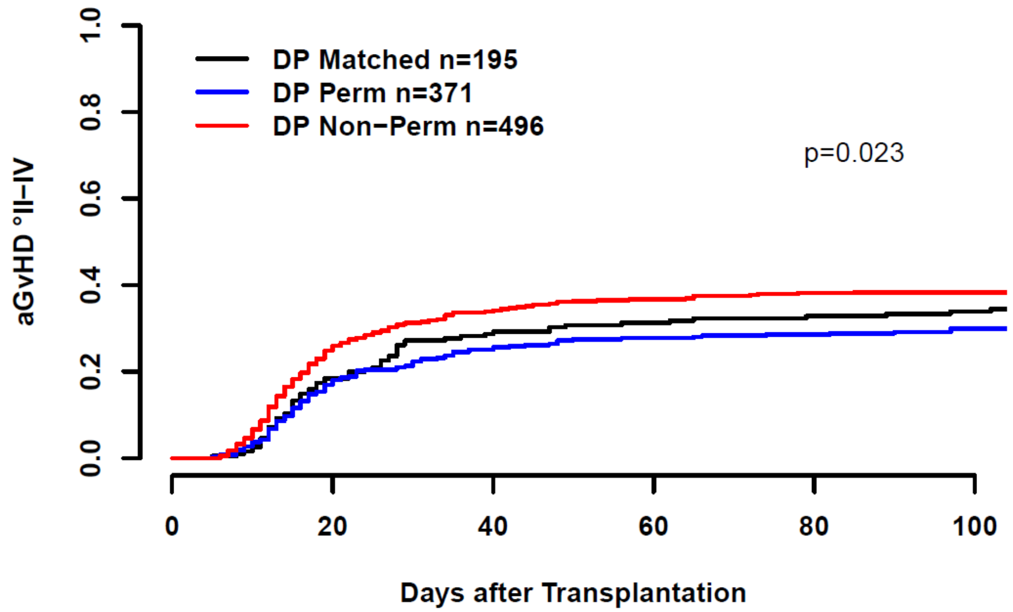

	0	20	40	60	80	100
DP Matched (n)	195	153	128	117	107	92
DP Perm (n)	371	298	253	226	207	185
DP Non−Perm (n)	496	360	295	266	243	227

Supp7(D): aGvHD II-IV in 9/10 HLA matched cases with respect to DP mismatch permissiveness according to TCE3. DP matched vs. DP Perm vs. DP Non-Perm cases, where DP Perm = TCE3 permissively mismatched and DP Non-Perm = DP TCE3 non-permissively mismatched (p=0.023).

Supp7 (E): 10/10, DP Matched vs. DP Perm. vs. DP Non−Perm.

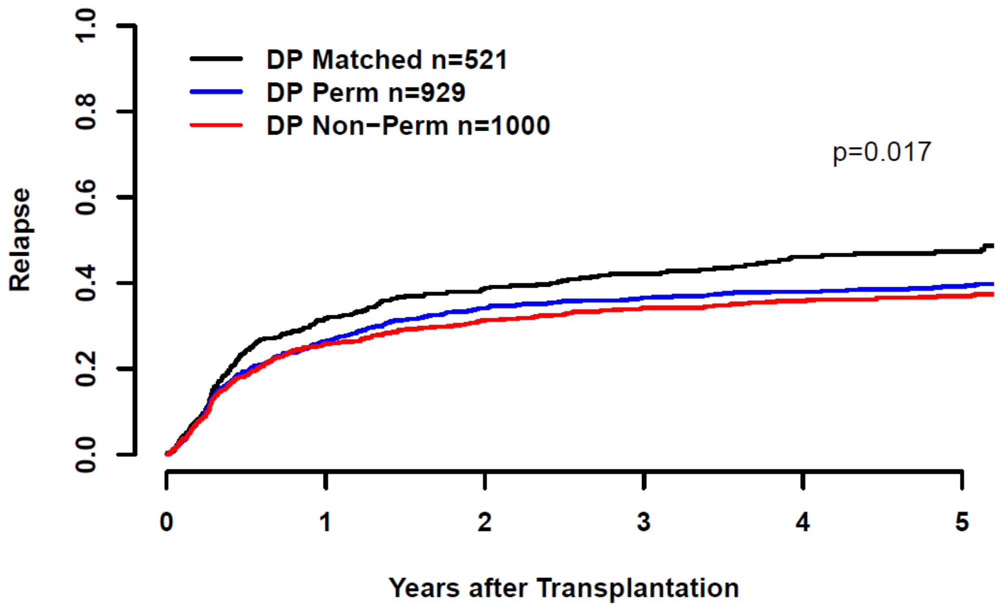

DP Matched (n)	521	203	114	71
DP Perm (n)	929	442	256	181
DP Non−Perm (n)	1000	421	256	168

Supp7(E): Relapse in 10/10 HLA matched cases with respect to DP mismatch permissiveness according to TCE3. DP matched vs. DP Perm vs. DP Non-Perm cases, where DP Perm = TCE3 permissively mismatched and DP Non-Perm = DP TCE3 non-permissively mismatched (p=0.017).

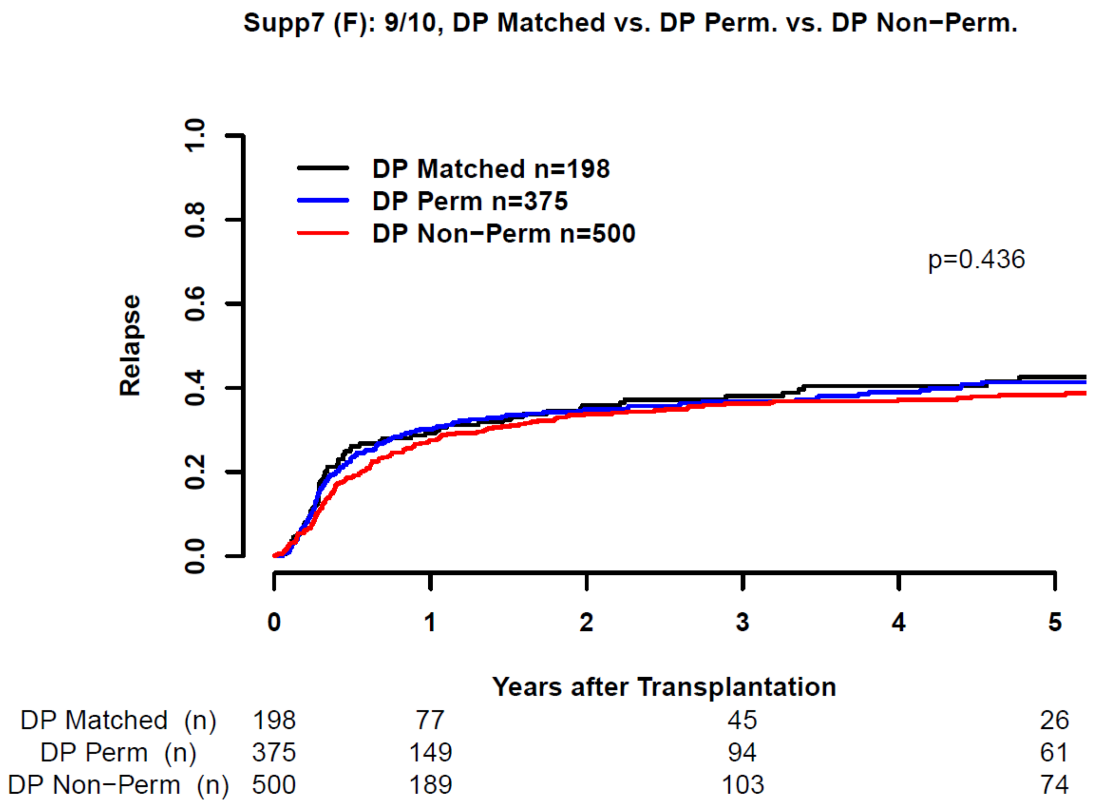

Supp7(D): Relapse in 9/10 HLA matched cases with respect to DP mismatch permissiveness according to TCE3. DP matched vs. DP Perm vs. DP Non-Perm cases, where DP Perm = TCE3 permissively mismatched and DP Non-Perm = DP TCE3 non-permissively mismatched (p=0.436).

Supp8: Cohort flowchart with regard to DP mismatch categorization

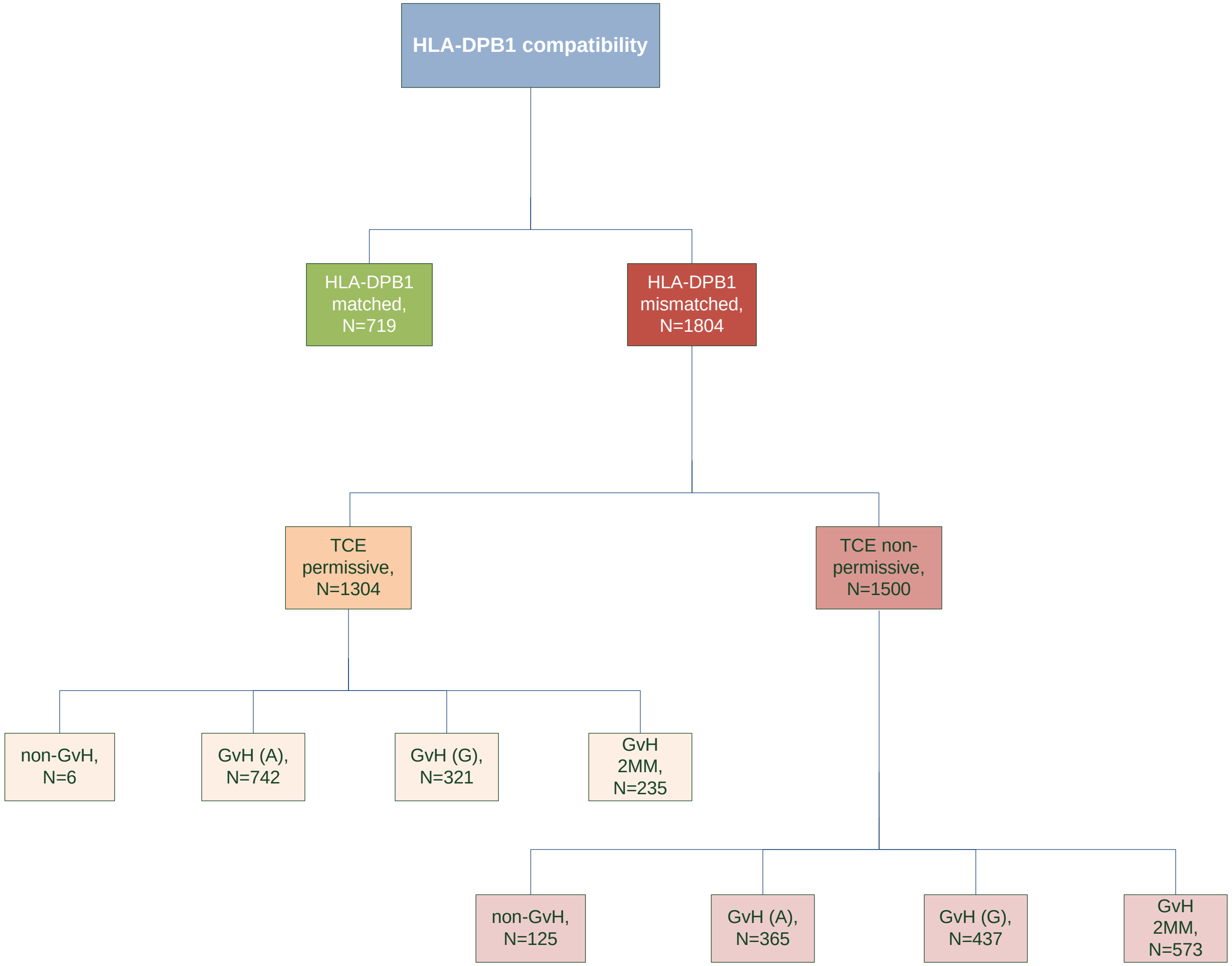

Table S1: Analysis of time-dependent variables

Endpoint	Variable	Time Period Post Tx	HR (CI)	P-value
OS	Advanced disease stage	Until 1 year	2.04 (1.77-2.35)	<0.001
		After 1 year	1.38 (1.10-1.72)	0.005
	MAC	Until 100 days	1.59 (1.31-1.94)	<0.001
		After 100 days	1.06 (0.92-1.22)	0.395
	In vivo T-cell depletion	Until 100 days	1.02 (0.84-1.24)	0.848
		After 100 days	0.81 (0.70-0.95)	0.007
GRFS	Patient age	Until 100 days	1.00 (1.00-1.01)	0.084
		After 100 days	1.02 (1.02-1.03)	<0.001
	In vivo T-cell depletion	Until 200 days	0.74 (0.66-0.82)	<0.001
		After 200 days	0.99 (0.82-1.19)	0.901
	TCE3 Non-permissive	Until 100 days	1.23 (1.11-1.36)	<0.001
		After 100 days	1.07 (0.94-1.22)	0.311
	TCE3 Non-permissive GvH	Until 100 days	1.31 (1.16-1.48)	<0.001
		After 100 days	1.03 (0.87-1.21)	0.757
NRM	Advanced disease stage	Until 1 year	1.72 (1.45-2.03)	<0.001
		After 1 year	1.03 (0.74-1.43)	0.878
	MAC	Until 100 days	1.69 (1.37-2.08)	<0.001
		After 100 days	0.99 (0.83-1.19)	0.939
REL	Advanced disease stage	Until 1 year	2.63 (2.23-3.11)	<0.001
		After 1 year	1.76 (1.38-2.25)	<0.001
	KPS<80	Until 200 days	2.02 (1.55-2.63)	<0.001
		After 200 days	0.97 (0.54-1.73)	0.916

Abbreviations: OS= Overall survival, GRFS=GvHD and relapse free survival, NRM=Non-relapse mortality, REL=Relapse, MAC=Myeloablative conditioning, TCE3=T-cell epitope 3 matching algorithm, KPS=Karnofsky Performance Score, Tx=Transplantation, HR=Hazard ratio, CI=Confidence interval.

Table S2: Multivariate Analysis aGvHD III-IV

	aGvHD III-IV	
	HR (CI)	P-value
**Patient Age**	-	
**Early stage disease**	-	
**Intermediate stage disease**	-	
**Advanced disease stage**	-	
**10/10 HLA**	1.00	
**9/10 HLA**	1.61 (1.32-1.96)	**<0.001**
**Patient HLA-C KIR Ligand group C1x**	-	
**Patient HLA-C KIR Ligand group C2C2**	-	
**Donor age 18-30**	1.00	
**Donor age 31-45**	1.38 (1.09-1.73)	**<0.001**
**Donor age 46-60**	1.12 (0.99-1.26)	**<0.001**
**P-D CMV neg-neg**	-	
**P-D CMV neg-pos**	-	
**P-D CMV pos neg**	-	
**P-D CMV pos pos**	-	
**RIC**	-	
**MAC**	-	
**KPS 80-100**	-	
**KPS <80**	-	
**No in-vivo T-cell depletion**	1.00	
**In vivo T-cell depletion**	0.62 (0.49-0.77)	**<0.001**
**Year of Tx 2000-2003**	1.00	
**Year of Tx 2004-2009**	0.58 (0.32-1.05)	0.073
**Year of Tx 2010-2017**	0.61 (0.33-1.13)	0.118
**TCE3 Permissive/DP matched**	1.00	
**TCE3 Non-permissive**	1.38 (1.14-1.67)	**<0.001**
**DP matched**	1.00	
**TCE3 permissive**	1.02 (0.77-1.34)	0.882
**TCE3 Non-permissive**	1.40 (1.08-1.81)	**0.011**
**TCE3 Permissive/DP matched**	1.00	
**TCE3 Non-permissive GvH**	1.55 (1.23-1.94)	**<0.001**
**TCE3 Non-permissive HvG**	1.23 (0.97-1.55)	0.088

Abbreviations: P-D Patient-Donor, RIC=Reduced intensity conditioning, MAC=Myeloablative conditioning, TCE3= T-cell epitope 3 matching, HR=Hazard ratio, CI=Confidence interval. - = not significant. Statistical significance marked in bold.

Table S3: Multivariate analysis of Relapse in advanced-disease-stage patients

	Relapse	
	HR (CI)	P-value
**Patient Age**	-	
**Early stage disease**	-	
**Intermediate stage disease**	-	
**Advanced disease stage**	-	
**10/10 HLA**	-	
**9/10 HLA**	-	
**Patient HLA-C KIR Ligand group C1x**	-	
**Patient HLA-C KIR Ligand group C2C2**	-	
**Donor age 18-30**	-	
**Donor age 31-45**	-	
**Donor age 46-60**	-	
**P-D CMV neg-neg**	-	
**P-D CMV neg-pos**	-	
**P-D CMV pos neg**	-	
**P-D CMV pos pos**	-	
**RIC**	-	
**MAC**	-	
**KPS 80-100**	1.00	
**KPS <80**	1.53 (1.13-2.05)	**0.005**
**No in-vivo T-cell depletion**	1.00	
**In vivo T-cell depletion**	0.80 (0.63-1.01)	0.062
**Year of Tx 2000-2003**	1.00	
**Year of Tx 2004-2009**	1.81 (0.90-3.63)	0.095
**Year of Tx 2010-2017**	2.15 (1.05-4.37)	0.035
**TCE3 Permissive/DP matched**	1.00	
**TCE3 Non-permissive**	0.83 (0.70-0.98)	**0.028**
**DP matched**	1.00	
**TCE3 permissive**	0.88 (0.71-1.11)	0.288
**TCE3 Non-permissive**	0.76 (0.61-0.96)	**0.02**
**TCE3 Permissive/DP matched**	1.00	
**TCE3 Non-permissive GvH**	0.83 (0.67-1.04)	0.112
**TCE3 Non-permissive HvG**	0.82 (0.67-1.01)	0.061

Abbreviations: P-D Patient-Donor, RIC=Reduced intensity conditioning, MAC=Myeloablative conditioning, TCE3= T-cell epitope 3 matching, HR=Hazard ratio, CI=Confidence interval. - = not significant. Statistical significance marked in bold.

Table S4: Death cause analysis

	GvHD	Infection	LPD	Other non TRM/REL	Poor Graft Function	Relapse/Progress	Sec.Mal	Toxicity	Unknown
**Non-permissive mismatch, N=1500**	60 (8.1%)	251 (34.1%)	3 (0.4%)	93 (12.6%)	2 (0.3%)	249 (33.8%)	12 (1.6%)	60 (8.1%)	7 (1.0%)
**Permissive mismatch, N=1304**	37 (6.2%)	190 (31.7%)	1 (0.2%)	63 (10.5%)	2 (0.3%)	249 (41.6%)	10 (1.7%)	42 (7.0%)	5 (0.8%)
**DP-matched, N=719**	24 (6.9%)	98 (28.1%)	0 (0.0%)	40 (11.5%)	0 (0.0%)	155 (44.4%)	3 (0.9%)	24 (6.9%)	5 (1.4%)

Abbreviations: LPD = Lymphoproliferative disease, Sec.Mal. = Secondary malignancy

Table S5: Multivariate analysis in 10/10 HLA matched cases

	OS		GRFS		NRM		aGVHD II-IV		Relapse	
	HR (CI)	P-value	HR (CI)	P-value	HR (CI)	P-value	HR (CI)	P-value	HR (CI)	P-value
**Patient Age**	1.02 (1.01-1.02)	**<0.001**	1.01 (1.00-1.01)*	**<0.001**	1.02 (1.01-1.03)	**<0.001**	-		1.01 (1.00-1.01)	**0.002**
**Early stage disease**	1.00		1.00		1.00		-		1.00	
**Intermediate stage disease**	1.24 (1.05-1.46)	**0.010**	1.30 (1.14-1.48)	**<0.001**	1.01 (0.82-1.24)	0.923	-		1.56 (1.33-1.84)	**<0.001**
**Advanced disease stage**	1.92 (1.65- 2.25)*	**<0.001**	1.57 (1.38-1.78)	**<0.001**	1.40 (1.16-1.68)*	**<0.001**	-		1.90 (1.62-2.24)*	**<0.001**
**Patient HLA-C KIR Ligand group C1x**	1.00		-		-		-		-	
**Patient HLA-C KIR Ligand group C2C2**	1.13 (0.96-1.34)	0.143	-		-		-		-	
**Donor age 18-30**	1.00		1.00		1.00		1.00		-	
**Donor age 31-45**	1.18 (1.03-1.36)	**0.015**	1.08 (0.97-1.21)	0.158	1.19 (1.01-1.41)	**0.043**	1.18 (0.99-1.42)	0.063	-	
**Donor age 46-60**	1.25 (1.04-1.51)	**0.018**	1.12 (0.97-1.30)	0.130	1.44 (1.15-1.79)	**0.001**	1.33 (1.05-1.69)	**0.016**	-	
**P-D CMV neg-neg**	1.00		-		-		-		-	
**P-D CMV neg-pos**	1.16 (0.92-1.47)	0.205	-		-		-		-	
**P-D CMV pos neg**	1.08 (0.91-1.28)	0.383	-		-		-		-	
**P-D CMV pos pos**	1.07 (0.92-1.25)	0.396	-		-		-		-	
**RIC**	1.00		1.00		1.00		-		-	
**MAC**	1.22 (1.05-1.41)*	**0.009**	1.11 (0.98-1.25)	0.099	1.27 (1.08-1.50)*	**0.005**	-		-	
**KPS 80-100**	1.00		1.00		-		-		1.00	
**KPS <80**	1.39 (1.05-1.85)	**0.023**	1.27 (1.01-1.59)	**0.039**	-		-		1.36 (1.03-1.79)*	**0.032**
**No in-vivo T-cell depletion**	1.00		1.00		-		1.00		1.00	
**In vivo T-cell depletion**	0.80 (0.67-0.95)*	**0.013**	0.79 (0.69-0.91)*	**0.001**	-		0.67 (0.56-0.80)	**<0.001**	0.84 (0.72-0.99)	**0.034**
**Year of Tx 2000-2003**	-		1.00		1.00		1.00		1.00	
**Year of Tx 2004-2009**	-		0.54 (0.36-0.82)	**0.004**	0.48 (0.30-0.77)	**0.002**	0.53 (0.29-0.97)	**0.039**	1.40 (0.79-2.48)	0.248
**Year of Tx 2010-2014**	-		0.53 (0.35-0.82)	**0.004**	0.45 (0.28-0.71)	**<0.001**	0.52 (0.28-0.96)	**0.036**	1.42 (0.79-2.54)	0.242
**TCE3 Permissive/DP matched**	1.00		1.00		1.00		1.00		1.00	
**TCE3 Non-permissive**	1.01 (0.89-1.14)	0.890	1.14 (1.03-1.26)*	**0.009**	1.19 (1.03-1.38)	**0.018**	1.33 (1.14-1.56)	**<0.001**	0.89 (0.79-1.01)	0.074
**DP matched**	1.00		1.00		1.00		1.00		1.00	
**TCE3 permissive**	0.93 (0.79-1.09)	0.393	0.96 (0.84-1.09)	0.546	0.99 (0.81-1.21)	0.938	1.13 (0.90-1.41)	0.289	0.85 (0.73-0.99)	**0.038**
**TCE3 Non-permissive**	0.96 (0.82-1.13)	0.656	1.11 (0.97-1.26)*	0.110	1.19 (0.98-1.44)	0.084	1.44 (1.17-1.78)	**<0.001**	0.81 (0.69-0.94)	**0.006**
**TCE3 Permissive/DP matched**	1.00		1.00		1.00		1.00		1.00	
**TCE3 Non-permissive GvH**	0.94 (0.80-1.10)	0.431	1.14 (1.01-1.29)*	**0.033**	1.15 (0.95-1.38)	0.153	1.47 (1.21-1.77)	**<0.001**	0.85 (0.72-0.99)	**0.038**
**TCE3 Non-permissive HvG**	1.08 (0.93-1.25)	0.330	1.13 (1.00-1.28)	**0.042**	1.24 (1.04-1.48)	**0.019**	1.21 (1.00-1.47)	**0.049**	0.94 (0.81-1.10)	0.455

Abbreviations: P-D Patient-Donor, RIC=Reduced intensity conditioning, MAC=Myeloablative conditioning, TCE3= T-cell epitope 3 matching, HR=Hazard ratio, CI=Confidence interval. Covariates showing time-dependent effects are labeled with an asterisk (*), Statistical significance marked in bold.

Table S6: Multivariate analysis in 9/10 HLA matched cases

	OS		GRFS		NRM		aGVHD II-IV		Relapse	
	HR (CI)	P-value	HR (CI)	P-value	HR (CI)	P-value	HR (CI)	P-value	HR (CI)	P-value
**Patient Age**	1.02 (1.01-1.03)	**<0.001**	1.01 (1.00-1.02)*	**<0.001**	1.03 (1.02-1.04)	**<0.001**	-		1.00 (0.99-1.01)	0.265
**Early stage disease**	1.00		1.00		1.00		-		1.00	
**Intermediate stage disease**	1.21 (0.97-1.51)	0.085	1.10 (0.92-1.32)	0.304	1.03 (0.80-1.32)	0.806	-		1.80 (1.40-2.30)	**<0.001**
**Advanced disease stage**	1.75 (1.41- 2.16)*	**<0.001**	1.45 (1.21-1.74)	**<0.001**	1.18 (0.92-1.50)*	0.185	-		2.37 (1.85-3.05)*	**<0.001**
**Patient HLA-C KIR Ligand group C1x**	1.00		-		-		-		-	
**Patient HLA-C KIR Ligand group C2C2**	1.32 (1.06-1.64)	**0.012**	-		-		-		-	
**Donor age 18-30**	1.00		1.00		1.00		1.00		-	
**Donor age 31-45**	1.04 (0.85-1.28)	0.685	1.09 (0.92-1.29)	0.320	1.16 (0.91-1.48)	0.221	1.14 (0.89-1.46)	0.308	-	
**Donor age 46-60**	1.28 (1.00-1.63)	**0.046**	1.11 (0.91-1.36)	0.303	1.61 (1.22-2.11)	**<0.001**	1.14 (0.85-1.53)	0.390	-	
**P-D CMV neg-neg**	1.00		-		-		-		-	
**P-D CMV neg-pos**	1.07 (0.78-1.46)	0.660	-		-		-		-	
**P-D CMV pos neg**	1.23 (0.98-1.55)	0.070	-		-		-		-	
**P-D CMV pos pos**	1.14 (0.91-1.44)	0.250	-		-		-		-	
**RIC**	1.00		1.00		1.00		-		-	
**MAC**	1.29 (1.05-1.59)*	**0.017**	1.26 (1.06-1.50)	**0.010**	1.32 (1.06-1.65)*	**0.013**	-		-	
**KPS 80-100**	1.00		1.00		-		-		1.00	
**KPS <80**	1.89 (1.36-2.62)	**<0.001**	1.57 (1.17-2.10)	**0.002**	-		-		1.37 (0.98-1.90)*	0.065
**No in-vivo T-cell depletion**	1.00		1.00		-		1.00		1.00	
**In vivo T-cell depletion**	0.90 (0.70-1.16)*	**0.415**	0.75 (0.61-0.93)*	**0.007**	-		0.69 (0.53-0.88)	**0.003**	0.71 (0.56-0.90)	**0.004**
**Year of Tx 2000-2003**	-		1.00		1.00		1.00		1.00	
**Year of Tx 2004-2009**	-		0.65 (0.42-1.01)	0.055	0.65 (0.39-1.08)	0.098	0.60 (0.37-0.97)	**0.035**	3.23 (1.25-8.39)	**0.016**
**Year of Tx 2010-2014**	-		0.68 (0.43-1.09)	0.107	0.73 (0.43-1.24)	0.244	0.65 (0.39-1.07)	0.090	3.27 (1.25-8.56)	**0.016**
**TCE3 Permissive/DP matched**	1.00		1.00		1.00		1.00		1.00	
**TCE3 Non-permissive**	1.05 (0.89-1.24)	0.573	1.19 (1.03-1.37)*	**0.015**	1.30 (1.07-1.58)	**0.007**	1.34 (1.09-1.65)	**0.004**	0.90 (0.75-1.08)	0.256
**DP matched**	1.00		1.00		1.00		1.00		1.00	
**TCE3 permissive**	0.87 (0.64-1.11)	0.262	0.90 (0.73-1.10)	0.311	0.93 (0.69-1.24)	0.612	0.99 (0.65-1.18)	0.395	0.99 (0.77-1.28)	0.930
**TCE3 Non-permissive**	0.96 (0.76-1.20)	0.703	1.11 (0.92-1.35)*	0.287	1.24 (0.95-1.62)	0.112	1.24 (0.94-1.63)	0.133	0.89 (0.69-1.15)	0.387
**TCE3 Permissive/DP matched**	1.00		1.00		1.00		1.00		1.00	
**TCE3 Non-permissive GvH**	0.95 (0.77-1.18)	0.647	1.24 (1.04-1.47)*	**0.016**	1.32 (1.04-1.67)	**0.022**	1.58 (1.24-2.03)	**<0.001**	0.78 (0.62-0.99)	**0.038**
**TCE3 Non-permissive HvG**	1.14 (0.94-1.39)	0.182	1.15 (0.97-1.36)	0.112	1.29 (1.02-1.62)	**0.030**	1.14 (0.89-1.47)	0.305	1.02 (0.83-1.27)	0.818

Abbreviations: P-D Patient-Donor, RIC=Reduced intensity conditioning, MAC=Myeloablative conditioning, TCE3= T-cell epitope 3 matching, HR=Hazard ratio, CI=Confidence interval.
Covariates showing time-dependent effects are labeled with an asterisk (*), Statistical significance marked in bold.

Table S7: cGvHD multivariate analysis in whole cohort

	cGvHD	
	**HR (CI)**	**P-value**
**Early stage disease**	1.00	
**Intermediate stage disease**	0.94 (0.79-1.11)	0.452
**Advanced disease stage**	0.85 (0.72- 1.02)*	0.075
**10/10 HLA**	1.00	
**9/10 HLA**	1.19 (1.04-1.36)	**0.009**
**Donor age 18-30**	1.00	
**Donor age 31-45**	1.13 (0.98-1.31)	0.084
**Donor age 46-60**	0.95 (0.78-1.16)	0.610
**RIC**	1.00	
**MAC**	0.86 (0.75-0.98)*	**0.029**
**KPS 80-100**	1.00	
**KPS <80**	1.28 (0.90-1.81)	0.174
**No in-vivo T-cell depletion**	1.00	
**In vivo T-cell depletion**	0.69 (0.59-0.80)*	**<0.001**
**TCE3 Permissive/DP matched**	1.00	
**TCE3 Non-permissive**	1.08 (0.95-1.23)	0.240
**DP matched**	1.00	
**TCE3 permissive**	1.03 (0.86-1.23)	0.771
**TCE3 Non-permissive**	1.10 (0.92-1.31)	0.296
**TCE3 Permissive/DP matched**	1.00	
**TCE3 Non-permissive GvH**	1.03 (0.88-1.21)	0.686
**TCE3 Non-permissive HvG**	1.12 (0.96-1.31)	0.136

Abbreviations: P-D Patient-Donor, RIC=Reduced intensity conditioning, MAC=Myeloablative conditioning, TCE3= T-cell epitope 3 matching, HR=Hazard ratio, CI=Confidence interval. Covariates showing time-dependent effects are labeled with an asterisk (*), - = not included in model. Statistical significance marked in bold.
